# Effect of Epidemic Intermittent Fasting on Cardiometabolic Risk Factors: A Systematic Review and Meta-Analysis of Randomized Controlled Trials

**DOI:** 10.3389/fnut.2021.669325

**Published:** 2021-10-18

**Authors:** Fan Yang, Can Liu, Xu Liu, Xiandu Pan, Xinye Li, Li Tian, Jiahao Sun, Shengjie Yang, Ran Zhao, Na An, Xinyu Yang, Yonghong Gao, Yanwei Xing

**Affiliations:** ^1^Guang'anmen Hospital, Chinese Academy of Chinese Medical Sciences, Beijing, China; ^2^Beijing University of Chinese Medicine, Beijing, China; ^3^Key Laboratory of Chinese Internal Medicine of the Ministry of Education, Dongzhimen Hospital Affiliated to Beijing University of Chinese Medicine, Beijing, China

**Keywords:** meta-analysis, intermittent fasting, weight loss, blood pressure, fasting blood glucose, blood lipid, cardiometabolic risk factors

## Abstract

Intermittent fasting (IF) has gained attention as a promising diet for weight loss and dysmetabolic diseases management. This systematic review aimed to investigate the effects of IF on cardiometabolic risk factors (CMRFs). A systematic literature search was carried out using three electronic databases, namely PubMed, Embase, and the Cochrane Library, until October 2020. Randomized controlled trials that compared the IF intervention with a control group diet were included. Fourteen effect sizes were expressed as weighted mean difference (WMD) using a fixed-effects model and 95% confidence intervals (CI). Compared to the ones within control groups, participants exposed to the IF intervention reduced their body weight (WMD, −1.78 kg; 95% CI, −2.21 to −1.35; *p* <0.05), waist circumference (WMD, −1.19 cm; 95% CI, −1.8 to −0.57; *p* <0.05), fat mass (WMD, −1.26 kg; 95% CI, −1.57 to −0.95; *p* <0.05), body mass index (WMD, −0.58 kg/m^2^; 95% CI, −0.8 to −0.37; *p* <0.05), systolic blood pressure (WMD, −2.14 mmHg; 95% CI: −3.54 to −0.73; *p* <0.05), diastolic blood pressure (WMD: −1.38 mmHg, 95% CI, −2.35 to −0.41, *p* <0.05), fasting blood glucose (WMD: −0.053 mmol/L; 95% CI: −0.105 to 0.001; *p* <0.05), fasting insulin (WMD, −0.8 mIU/L; 95% CI, −1.15 to −0.44; *p* <0.05), insulin resistance (WMD, −0.21; 95% CI, −0.36 to −0.05; *p* <0.05), total cholesterol (WMD, −0.10 mmol/L; 95% CI, −0.17 to −0.02; *p* <0.05), and triglycerides (WMD, −0.09 mmol/L; 95% CI, −0.13 to −0.04; *p* <0.05). No effects were observed for low-density lipoprotein cholesterol, high-density lipoprotein cholesterol, or glycosylated hemoglobin. This meta-analysis supports the role of IF in improving the component composition of CMRFs, including weight, waist circumference, fat mass, BMI, blood pressure, total cholesterol, triglycerides, fasting insulin, and insulin resistance, compared to a control group diet. Further research on IF interventions should take into account long-term and well-designed administration to draw definitive conclusions.

## Introduction

The term “cardiometabolic risk” was first employed by the American Diabetes Association (1) as an umbrella term to include all risk factors for diabetes and cardiovascular disease (CVD) (2). Cardiometabolic risk factors (CMRFs), including obesity, hypertension, diabetes mellitus, and hyperlipidemia, are major contributors to atherosclerosis, ischemic heart disease, stroke, and certain cancers (3). The worldwide burden of cardiometabolic risk has expanded rapidly (4). Mortality from cardiometabolic diseases such as type 2 diabetes mellitus, CVD, and coronary heart disease has increased, and CVD is now the leading cause of death (5). Calorie restriction is an effective management strategy for CMRFs (6). Intermittent fasting (IF), an important measure of energy restriction, also significantly improves metabolic disease (7–10).

IF has gradually come into focus in our daily lives (11). At present, a large number of studies have shown that IF is beneficial in the treatment of metabolic diseases, as well as dietary intervention is easier to accept than drug treatment. Dietary restrictions (12) through IF have been shown to improve metabolic disease risk indicators. Further, IF reportedly plays a considerable role in regulating cardiovascular risk indicators (13), insulin resistance (HOMA-IR), and circulating blood glucose levels (14, 15). There are different types of IF that act as an energy-limiting diet for a specific period, including alternate-day fasting (ADF), alternate-modified-day fasting (AMDF), periodic fasting (PF), time-restricted feeding (TRF), and religious fasting (Table 1). Intermittent energy restriction (IER) is an alternative of IF in this study, and the control group is often a continuous energy restriction (CER).

**Table 1:** Types of intermittent fasting and description.

**Type of fast**	**Description**
Alternate-day fasting (ADF)	A circular diet requires fasting for a day (consumption of no calories) and then eating freely for a day (1).
Alternate-modified-day fasting (AMDF)	A circular feeding pattern that requires fasting (consumption of 20–25% of energy needs) for a day, and then eating freely for a day; the popular 5:2 diet includes a discontinuous strict energy limit of 2 days a week and 5 other days of random eating (16, 17).
Time-restricted feeding (TRF)	Complete fast (no calories) for at least 12 h a day, and eating freely the rest of the time; the 16:8 fasting pattern currently prevails (9, 16, 18).
Periodic fasting (PF)	A circular weekly eating pattern that consists of fasting 1 to 2 days a week (burning 25% or less of the calories required) and eating freely the rest of the week on a 6:1 or 5:2 scale (9).
Common religious fasts	These include: 1. The Islamic Ramadan fast: during the 30-day fasting holy month of Ramadan, worshippers fast from sunrise to sunset and eat freely after sunset (19). 2. Greek Orthodox fasts: During fasting, people fast dairy products, eggs, and meat for 40 days (20). 3. Daniel fast: This is a biblical fast, usually for 10–40 days (20). 4. Jewish fast: one of the major fasts in the Jewish calendar is the Yom Kippur fast (19).

Over the past three decades, original studies (16, 21) have investigated the impact of IF on a variety of health outcomes, including metabolic disease risk factors, such as weight, blood pressure (BP), waist circumference (WC), body fat, lipid distribution, and blood glucose. However, a recent study by Lowe et al. (22) demonstrated that in the absence of controlled food intake, IF will not play a significant role in weight loss. In turn, it will lead to a reduction in muscle mass. In some randomized crossover trials, IF had no effect on glucose and lipid metabolism (23, 24). These results indicate that the effects of IF on various metabolic factors are contradictory. Therefore, we need a comprehensive and systematic meta-analysis representing all included randomized controlled trials (RCTs), a large sample size, a variety of IF types, and multiple effect indicators to determine the effectiveness of IF interventions in improving health outcomes and modifiable risk factors for people with CMRFs.

## Methods

This study used a systematic review and a meta-analysis of PRISMA's preferred reporting items as a guide for reporting research results (25, 26).

### Data Source and Search Strategy

Articles were identified by searching through three electronic databases, i.e., PubMed, Embase, and the Cochrane Library until October 2020. Two reviewers (C.L. and X.L.) independently evaluated articles' eligibility, and the inconsistencies shall be made by the corresponding author (F.Y.). The search strategy is described in detail in the Supplementary Table 1.

### Inclusion and Exclusion Criteria

The articles had the following characteristics: (1) Type study: RCTs; (2) Participants: participants >18 years; (3) Intervention: different types of IF, including PF, ADF, AMDF, TRF, and part of IER; and (4) Outcomes: data on at least one CMRFs component: body composition (weight, WC, fat mass [FM], and body mass index [BMI]), BP (systolic blood pressure [SBP], diastolic blood pressure [DBP]), lipid panel (total cholesterol [TC], triglycerides (TG), low-density lipoprotein cholesterol [LDL-C], and high-density lipoprotein cholesterol [HDL-C]), glycemic control (fasting blood glucose [FBG], fasting insulin [Fins], glycosylated hemoglobin [HbA1c], and HOMA-IR).

The exclusion criteria were as follows: (1) uncontrolled trials or other study designs; (2) studies lacking a control group; (3) studies without CMRFs component as an outcome and/or lacking sufficient information; (4) non-human samples, reviews, case studies, as well as unpublished abstracts; (5) studies with animal models; (6) pregnant or lactating women; (7) studies in languages other than English; (8) absence of time limits in IER and fasting.

### Data Extraction and Study Quality Assessment

Two investigators independently extracted the relevant data from the eligible studies using predesigned forms. Data included study, publication year, country, study design, inclusion and exclusion criteria, total number of participants, participant details, study duration, intervention details and control groups, baseline patient characteristics (mean age, sex), body composition, BP, glycemic control, and lipid panel. Disagreements were resolved by consensus. When necessary, we emailed the corresponding author to acquire study details.

### Quality Assessment and Publication Bias

The researchers used Cochrane Collaboration's bias risk tool to evaluate the quality of the methodology included in the studies. According to the criteria of the Cochrane handbook for systematic reviews, the bias risk of each item is classified as low, high, or unclear (27).

### Data Statistical and Analysis

Effect estimates were expressed as weighted mean differences (WMD) with a 95% confidence interval (CI). Inter-study heterogeneity was tested using the Higgins *I*^2^ statistic, and *I*^2^ > 50% indicated significant statistical heterogeneity. The heterogeneity of the study and measurement of effect estimates were determined using the mean and standard deviation (SD) of the differences before and after IF intervention. Publication bias was evaluated using funnel plots; formal testing was conducted with Egger's test (28), and a sensitivity analysis was also performed. We used STATA 16 (StataCorp LLC, College Station, TX, USA) for the statistical analyses.

In order to determine the influence of IF on various effect indicators, it is necessary to change the mean value before and after intervention as well as the SD of the changes. Therefore, we used a method outlined in the Cochrane handbook (27, 29) to determine the SD of changes between time points. SD_change_ = √[SD${}_{\text{baseline}}^{2}+$ SD${}_{\text{final}}^{2}$- (2 × R × SD_baseline_× SD_final_)]. In addition, we performed some conversion of data units through international calculation formulas to ensure that results are clinically significant.

## Results

### Characteristics of Included Studies

**Figure 1:**
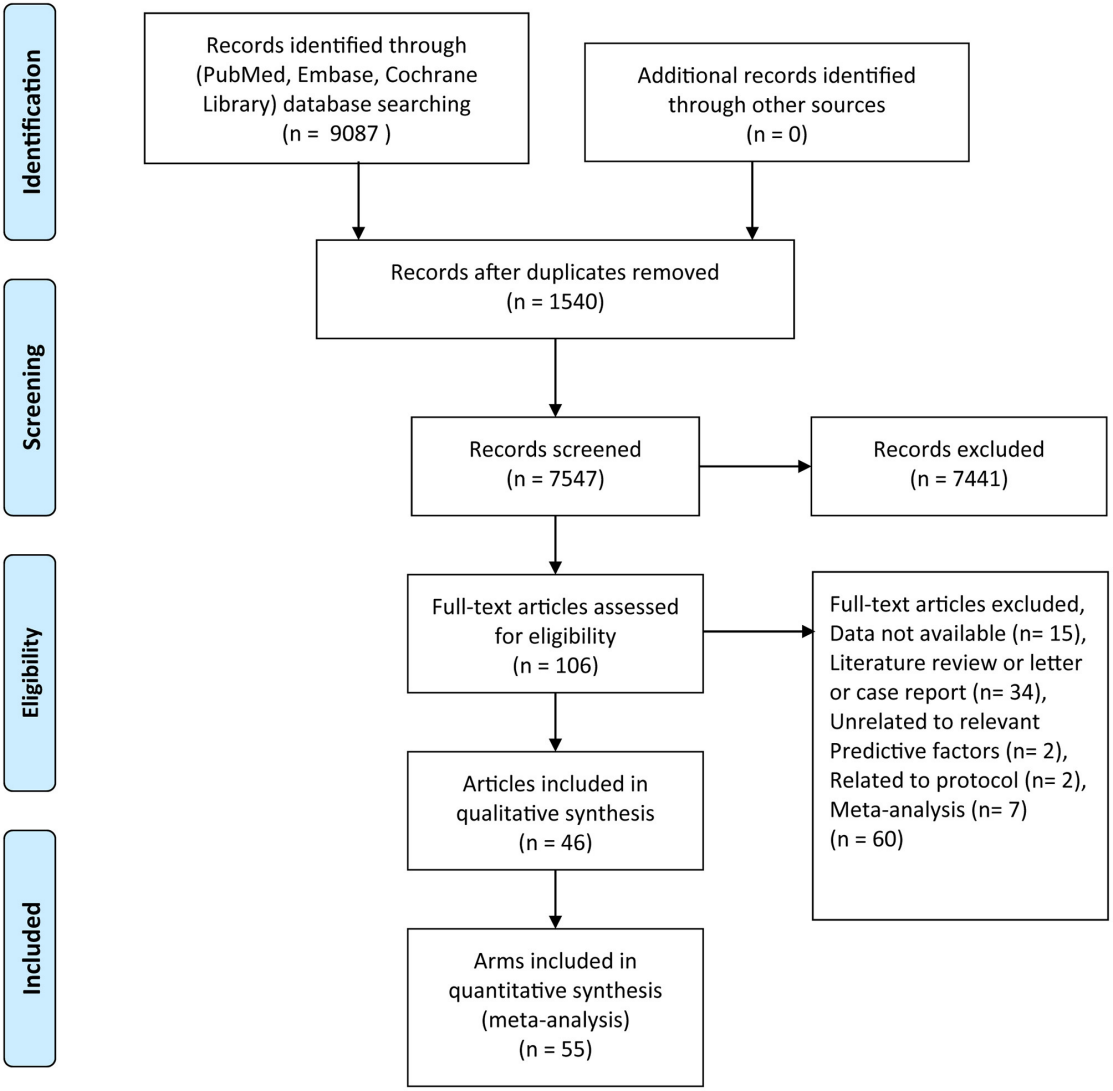
Schema of the search strategy.

The PRISMA statement flow diagram is shown in Figure 1 (30). A total of 9,087 articles were part of the initial database search (PubMed: 6128, EMBASE: 40, Cochrane Library: 2919), after the removal of 1,540 duplicate articles. After filtering the titles and abstracts to exclude irrelevant articles, we found 106 articles that met the topic of interest. The full texts of the 106 records were reviewed. Of them, 60 records were excluded for the following reasons: data not available (*n* = 15), literature review, letter, or case report (*n* = 34), unrelated to relevant predictive factors (*n* = 2), related to protocol (*n* = 2), and meta-analyses (*n* = 7). Finally, 46 articles from the database searches were included in the meta-analysis (12, 22, 31–75). A total of 2,681 participants were randomized in the IF intervention group (*n* = 1,423) and the control group (*n* = 1,258). The characteristics of the eligible 46 articles with 55 arms are summarized in Supplementary Table 2. All the results calculated using Stata are shown in Table 2.

**Table 2:** All the results calculated using Stata.

**Characteristic**	**Trials**	**Participants**	**WMD**	**95% CI**	***Z***	***P***	***I*^**2**^ (%)**	***P* for heterogeneity**
**Body composition**
Weight (kg)	45	2,225	−1.78	(−2.21, −1.35)	8.11	0.000	0	0.960
WC (cm)	23	1,385	−1.19	(−1.80, −0.57)	3.77	0.000	23.8	0.148
FM (kg)	33	1,610	−1.26	(−1.57, −0.95)	7.87	0.000	22.9	0.121
BMI (kg/m^2^)	26	1,590	−0.58	(−0.80, −0.37)	5.24	0.000	0	0.886
**Glycemic control**
FBG (mmol/L)	34	1,863	−0.053	(−0.105, −0.001)	2.02	0.044	44.4	0.003
Fins (mIU/L)	26	1,161	−0.80	(−1.15, −0.44)	4.40	0.000	24.3	0.130
HbA1c (%)	9	544	−0.06	(−0.18, 0.05)	1.07	0.287	0	0.974
HOMA–IR	19	866	−0.21	(−0.36, −0.05)	2.66	0.008	38.4	0.046
**Blood pressure**
SBP (mmHg)	29	1,393	−2.14	(−3.54, −0.73)	2.97	0.003	36.2	0.028
DBP (mmHg)	27	1,277	−1.38	(−2.35, −0.41)	2.79	0.005	0	0.588
**Lipid panel**
TC (mmol/L)	33	1,766	−0.10	(−0.17, −0.02)	2.54	0.011	14.6	0.233
TG (mmol/L)	34	1,750	−0.09	(−0.13, −0.04)	3.78	0.000	5.5	0.377
LDL–C (mmol/L)	36	1,850	−0.056	(−0.114, 0.003)	1.85	0.064	0	0.967
HDL–C (mmol/L)	36	1,852	−0.01	(−0.04, 0.01)	1.15	0.250	0	0.858

*WC, waist circumference; FM, fat mass; BMI, body mass index; SBP, systolic blood pressure; DBP, diastolic blood pressure; TC, total cholesterol; TG, triglyceride; HDL, high-density lipoprotein; LDL, low-density lipoprotein; FBG, fasting blood glucose; Fins, fasting insulin; HbA1c, glycosylated hemoglobin; HOMA-IR, insulin resistance*.

### Risk of Bias and Quality Assessment of Studies

**Figure 2:**
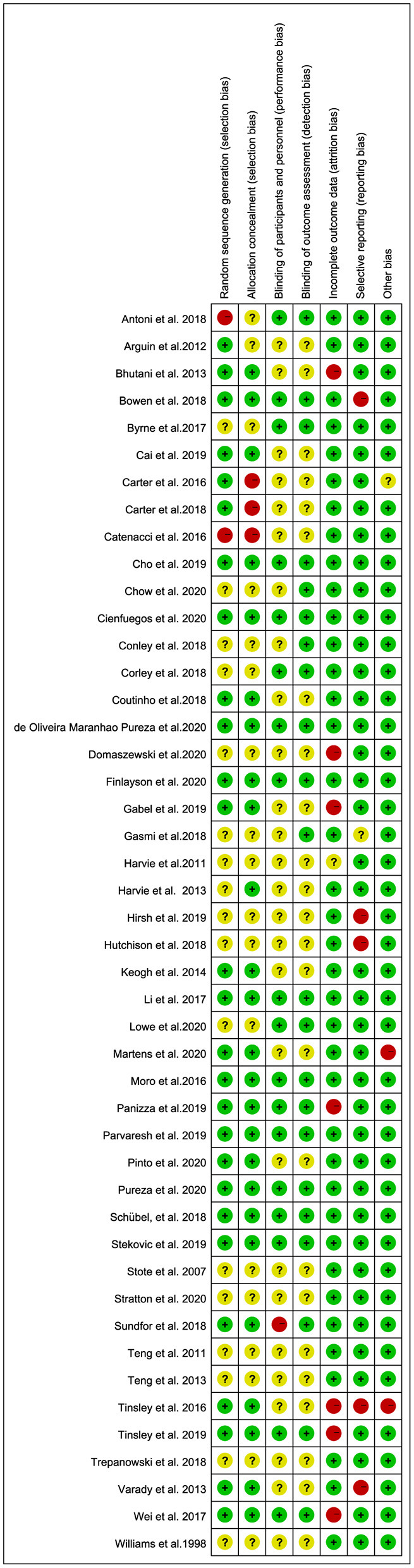
Risk-of-bias assessment of the studies included in the meta-analysis.

Figure 2 summarizes the risk of bias for RCTs. Twenty-six articles (56%) had a low risk of selection bias. This was because of the intervention type, as no RCT adequately performed blinding of the participants (blinding of dietary interventions is impossible); however, 22 articles (48%) were judged as having a low risk of bias for outcome assessment blinding, 38 articles (82%) were judged as having a low risk of bias for incomplete outcome data, 40 articles (86%) were judged as having a low risk of bias for selective reporting, and 43 articles (93%) showed a low risk of other biases. Overall, 17 articles (37%) were rated with a high risk of bias due to random sequence generation, allocation concealment, outcome assessment blinding, incomplete outcome data, and selective reporting.

### Meta-Analysis Results

#### Effect of IF on Body Composition

**Figure 3:**
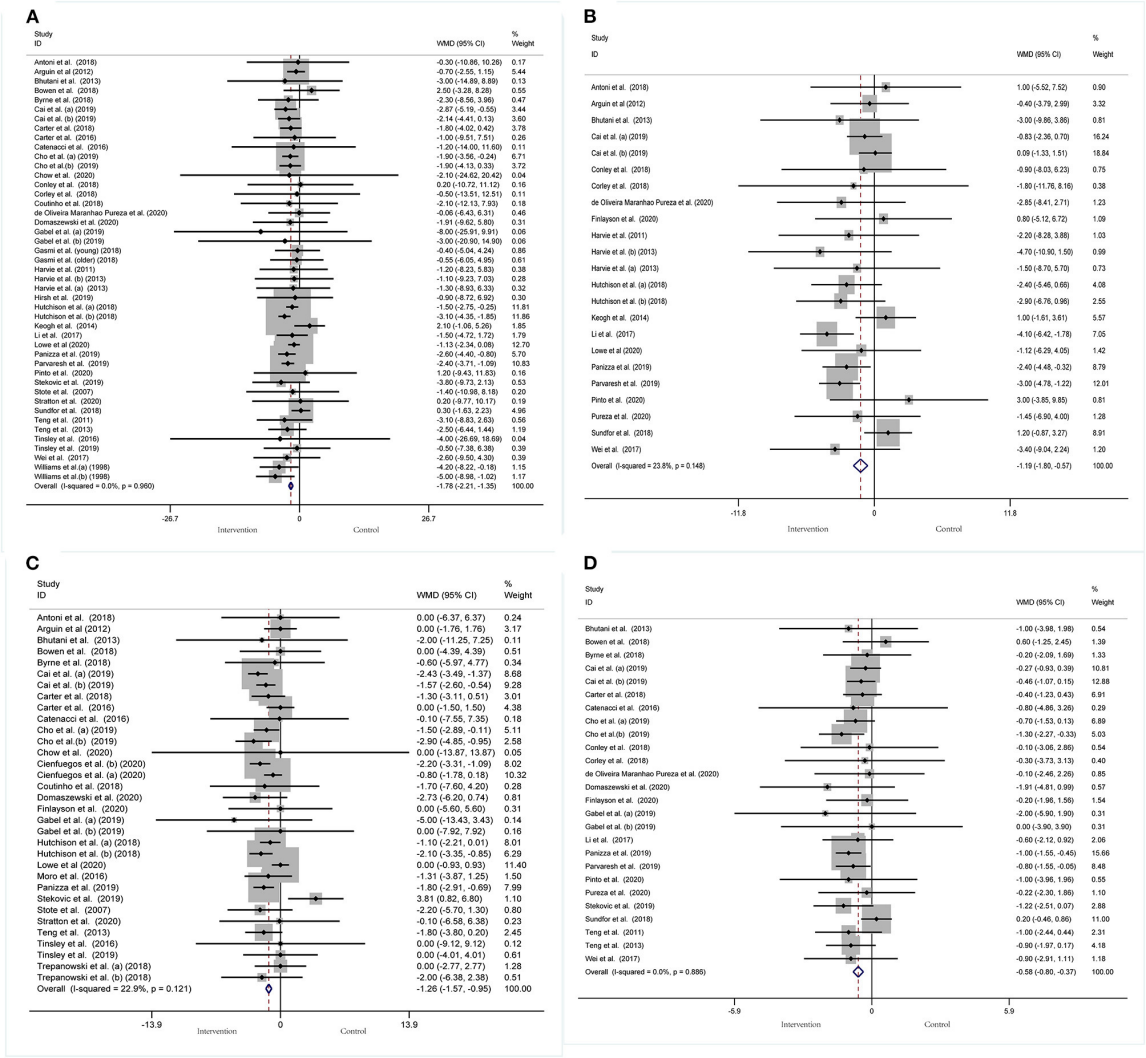
Forest plot of RCTs investigating the effects of intermittent fasting on body composition **(A)** Weight, **(B)** WC, **(C)** FM, **(D)** BMI.

**Figure 4:**
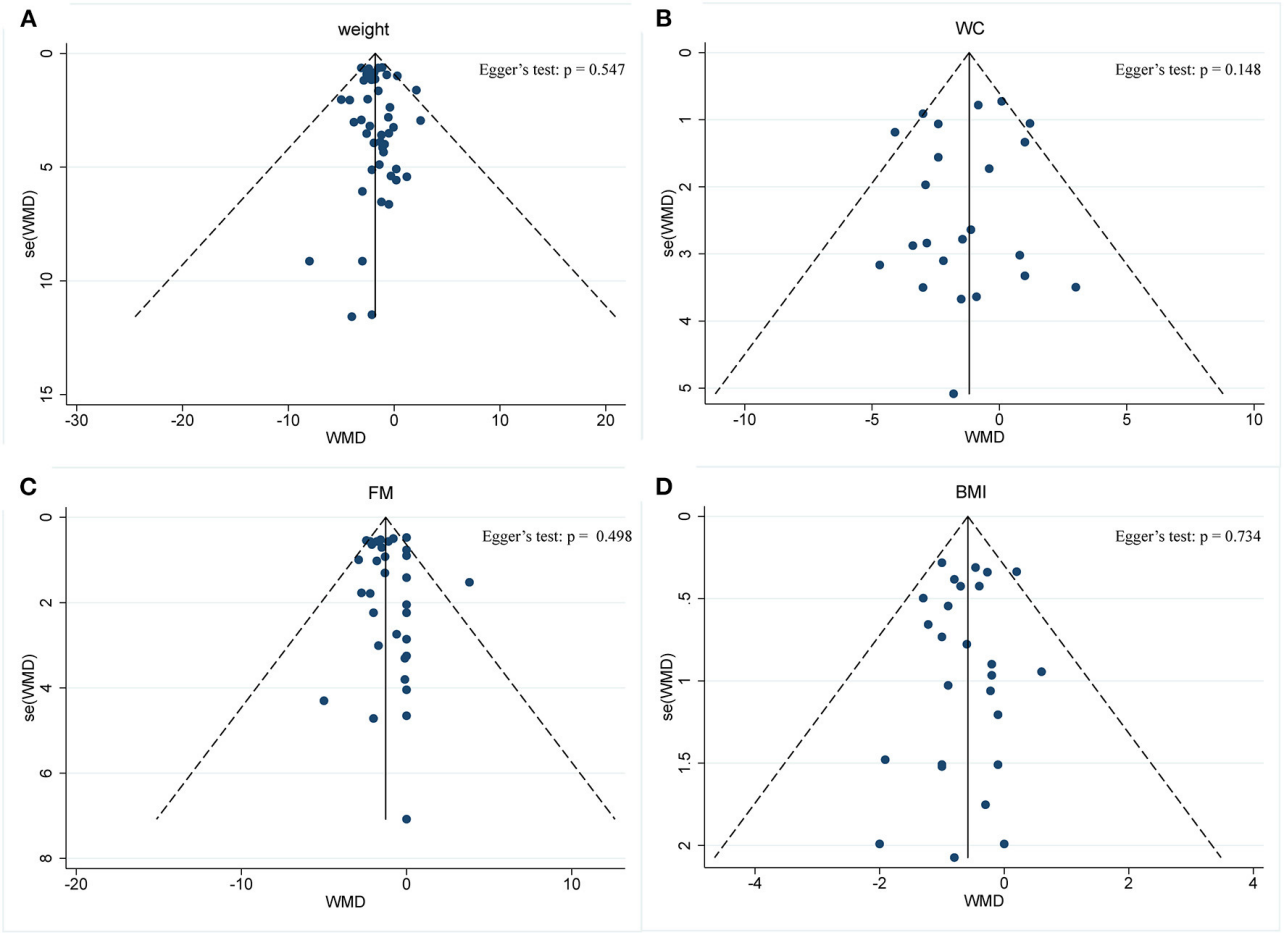
Funnel plot displaying no publication bias in the studies reporting the impact of intermittent fasting on body composition **(A)** Weight, **(B)** WC, **(C)** FM, **(D)** BMI.

Body composition was operationalized in weight, WC, FM, and BMI. Forty-five arms, with 2,225 participants (case = 1,136, control = 1,089), showed a consistent effect of IF on weight (Figure 3A). The fixed-effect analysis showed significant weight reduction (WMD, −1.78 kg, 95% CI: −2.21 to −1.35, *p* <0.05), thus, indicating significant weight loss. There was no evidence of effect heterogeneity (*I*^2^ = 0.0%, *p* = 0.96). Regarding funnel plot symmetry and Egger's test, *p* = 0.547 (Figure 4A).

Pooled data from 23 arms (1,385 participants: case = 714, control = 671) showed a consistent effect of IF on WC (Figure 3B). While the fixed-effect analysis showed a significant WC reduction (WMD: −1.19 cm, 95% CI: −1.8 to −0.57, *p* <0.05), there was also evidence of effect heterogeneity (*I*^2^ = 23.8%, *p* = 0.148). Regarding funnel plot symmetry and Egger's test, *p* = 0.576 (Figure 4B).

A pooled meta-analysis including 33 arms with 1,610 participants, found a significant effect of IF on FM when compared to placebo (WMD: −1.26 kg, 95% CI: −1.57 to −0.95, *p* <0.05) (Figure 3C). There was a slight effect heterogeneity (*I*^2^ = 22.9%, *p* = 0.121). Regarding funnel plot symmetry and Egger's test, *p* = 0.498 (Figure 4C).

The effects of IF on changes in BMI were assessed in 26 RCTs with 1,590 participants (case = 806, control = 784). The results showed a significant effect on the BMI (a fixed-effects model, WMD: −0.58 kg/m^2^, 95% CI: −0.8 to −0.37, *p* <0.05) (Figure 3D). There was no evidence of effect heterogeneity (*I*^2^ = 0.0%, *p* = 0.886). Regarding funnel plot symmetry and Egger's test, *p* = 0.734 (Figure 4D).

#### Effect of IF on Glycemic Control

**Figure 5:**
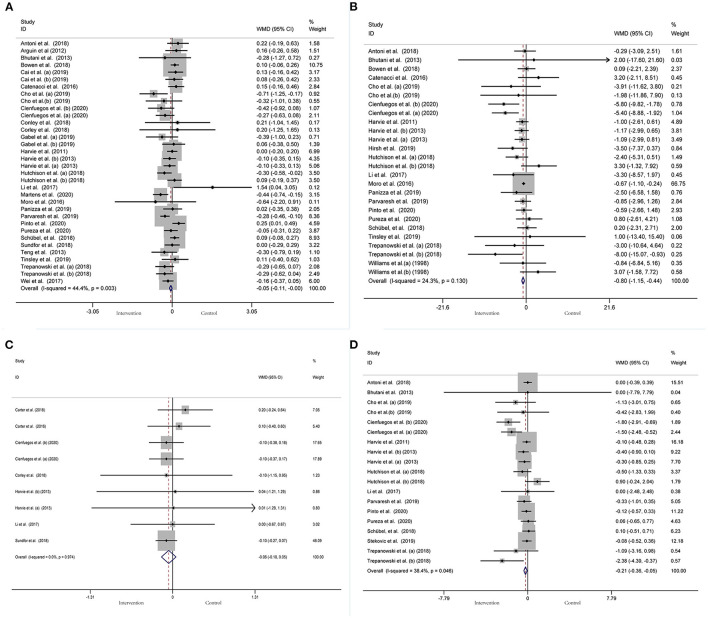
Forest plot of RCTs investigating the effects of intermittent fasting on glycemic control **(A)** FBG, **(B)** Fins, **(C)** HbA1c, **(D)** HOMA-IR.

**Figure 6:**
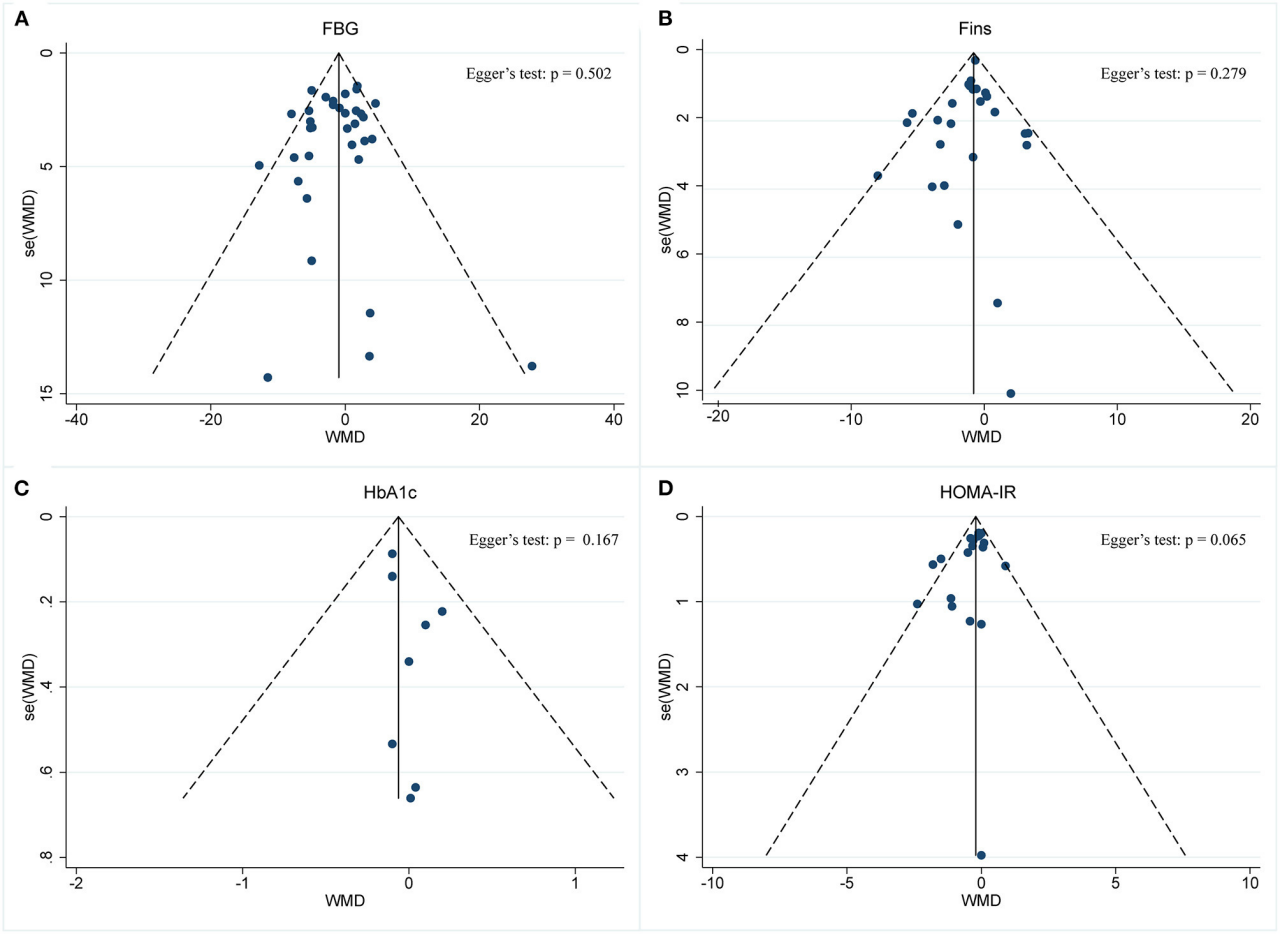
Funnel plot displaying no publication bias in the studies reporting the impact of intermittent fasting on glycemic control **(A)** FBG, **(B)** Fins, **(C)** HbA1c, **(D)** HOMA-IR.

A meta-analysis of the effect of IF on glycemic control was performed, including the relevant studies. A cumulative meta-analysis of 34 arms with 1,863 participants (case = 947, control = 916) evaluated changes in FBG during IF (Figure 5A). The WMD was −0.053 mmol/L (95% CI: −0.105 to −0.001, *p* <0.05, a fixed-effects model), which indicates significant FBG reduction. We observed a moderate effect heterogeneity (*I*^2^ = 44.4%, *p* = 0.003). Regarding funnel plot symmetry and Egger's test, *p* = 0.502 (Figure 6A).

A pooled meta-analysis including 26 arms with 1,160 participants (case = 584, control = 576) showed significant Fins reduction (WMD: −0.8 mIU/L, 95% CI: −1.15 to −0.44, *p* <0.05) (Figure 5B). There was evidence of effect heterogeneity (*I*^2^ = 24.3%, *p* = 0.13). Regarding funnel plot symmetry and Egger's test, *p* = 0.279 (Figure 6B).

A pooled meta-analysis including nine arms with 544 participants (case = 280, control = 264) reported changes in HbA1c during IF (Figure 5C). The WMD was −0.06% (95% CI: −0.18 to 0.05, *p* > 0.05, a fixed-effects model), which indicates no tangible effect in HbA1c. There was no evidence of effect heterogeneity (*I*^2^ = 0.0%, *p* = 0.974). Regarding funnel plot symmetry and Egger's test, *p* = 0.167 (Figure 6C).

Pooled data from 19 arms with 866 participants (case = 443, control = 423) reported the effect of IF on HOMA-IR (Figure 5D). The WMD using a fixed-effects model was −0.21 (95% CI: −0.36 to −0.05, *p* <0.05), which indicates significant HOMA-IR reduction. We observed a mild level of heterogeneity among the studies (*I*^2^ = 38.4%, *p* = 0.046). Regarding funnel plot symmetry and Egger's test, *p* = 0.065 (Figure 6D).

#### Effect of IF on BP

**Figure 7:**
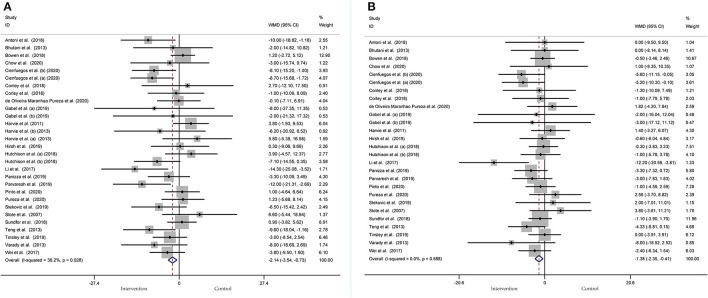
Forest plot of RCTs investigating the effects of intermittent fasting on blood pressure **(A)** SBP and **(B)** DBP.

**Figure 8:**
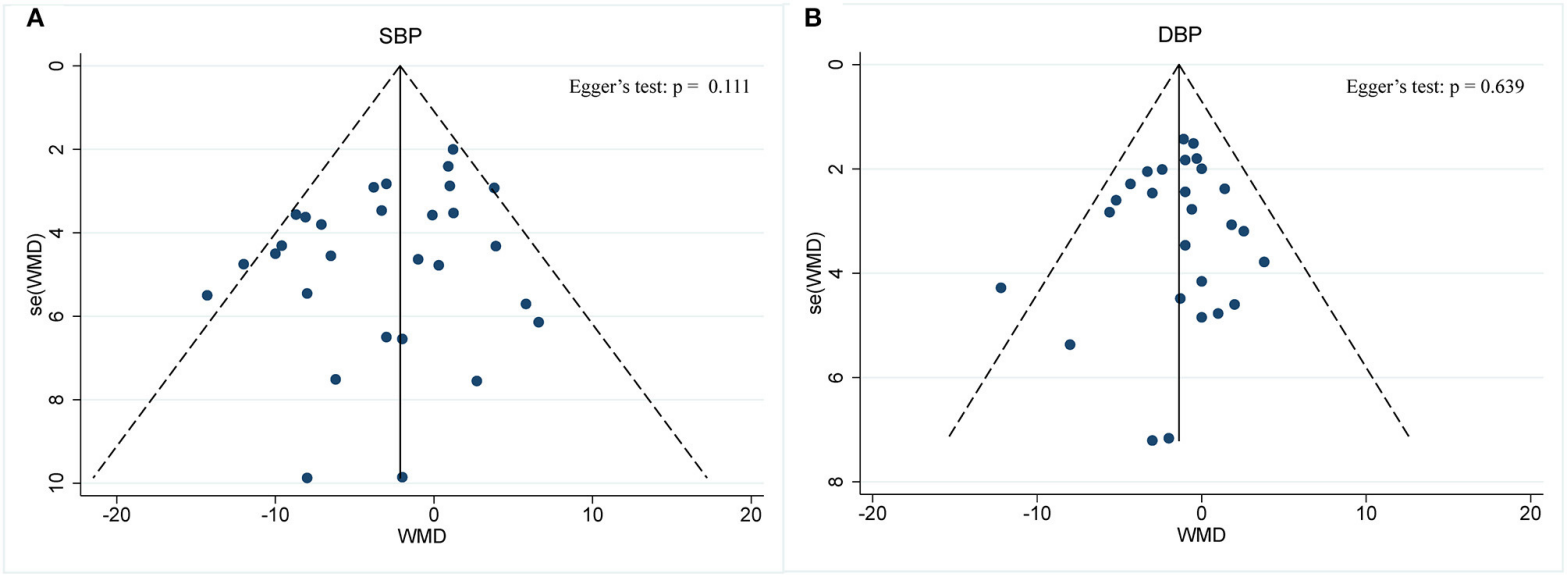
Funnel plot displaying no publication bias in the studies reporting the impact of intermittent fasting on blood pressure **(A)** SBP and **(B)** DBP.

BP was operationalized in SBP and DBP. In a pooled meta-analysis including 29 arms with 1,393 participants, we found a tangible effect of IF on SBP level when compared to placebo (a fixed-effects model, WMD: −2.14 mmHg, 95% CI: −3.54 to −0.73, *p* <0.05) (Figure 7A). We found mild effect heterogeneity (*I*^2^ = 36.2%, *p* = 0.028). Regarding funnel plot symmetry and Egger's test, *p* = 0.111 (Figure 8A).

Twenty-seven arms with 1,277 participants (case = 640, control = 637) indicated an IF effect on DBP (Figure 7B). The WMD using a fixed-effects model was −1.38 mmHg (95% CI: −2.35 to −0.41, *p* <0.05), which indicates significant DBP reduction. There was no evidence of effect heterogeneity among studies (*I*^2^ = 0.0%, *p* = 0.588). Regarding funnel plot symmetry and Egger's test, *p* = 0.639 (Figure 8B).

#### Effect of IF on Blood Lipid Panel

**Figure 9:**
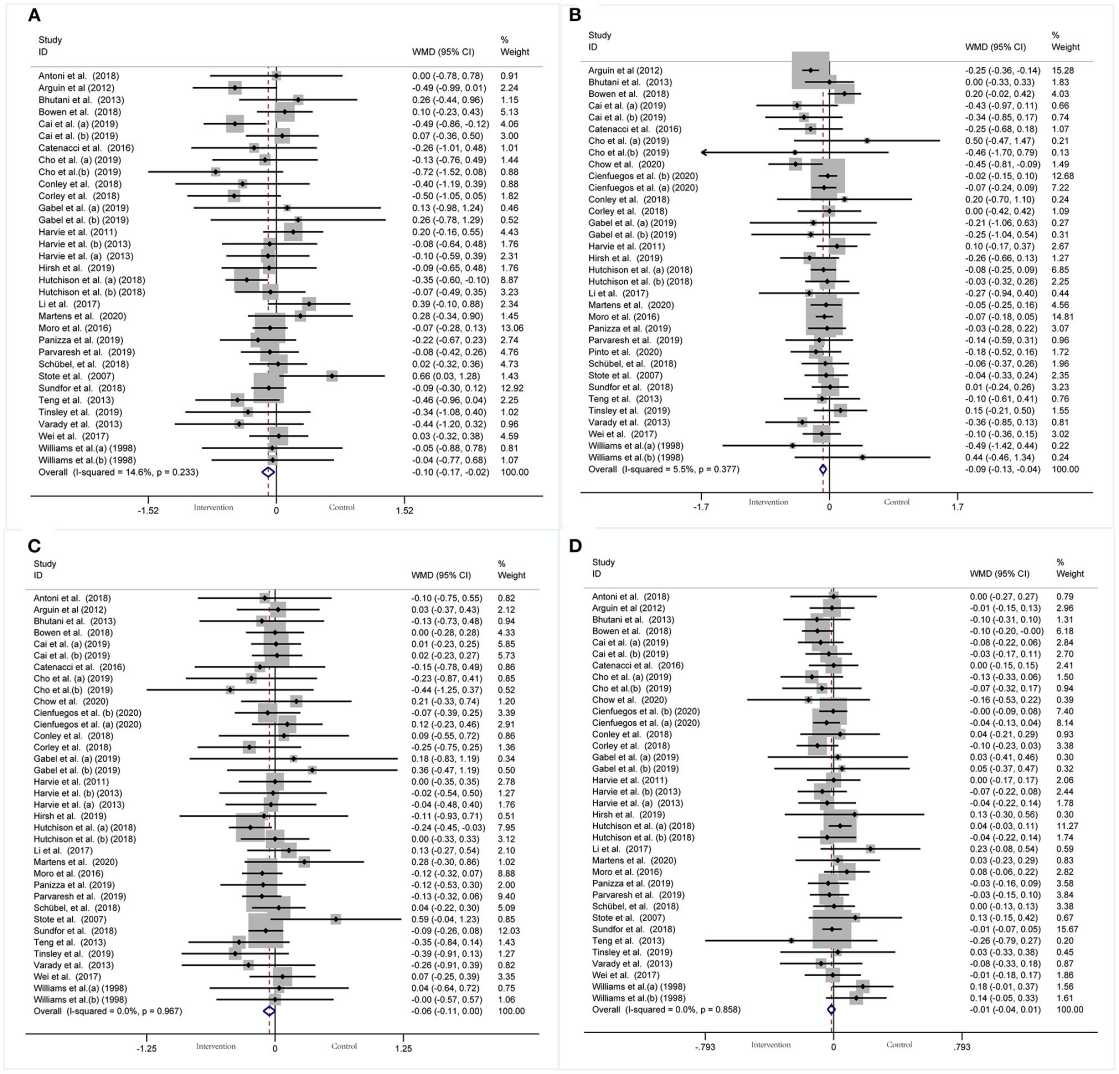
Forest plot of RCTs investigating the effects of intermittent fasting on lipid panel **(A)** TC, **(B)** TG, **(C)** LDL-C, **(D)** HDL-C.

**Figure 10:**
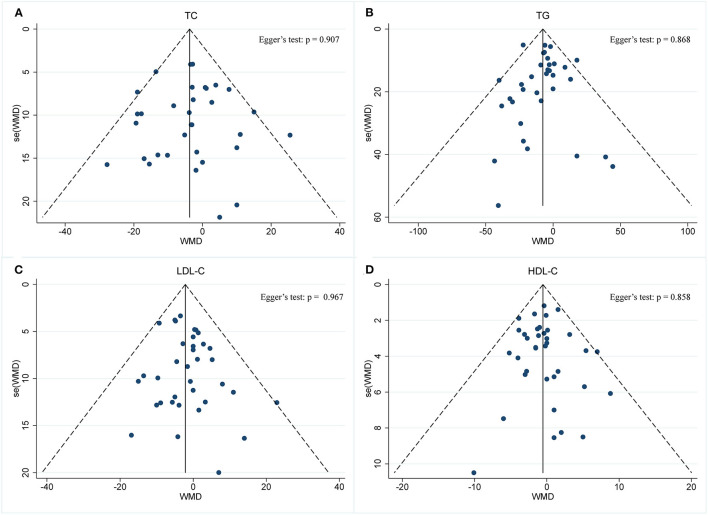
Funnel plot displaying no publication bias in the studies reporting the impact of intermittent fasting on lipid panel **(A)** TC, **(B)** TG, **(C)** LDL-C, **(D)** HDL-C.

A meta-analysis of blood lipid levels was performed involving TC, TG, HDL-C, and LDL-C. In 33 arms with 1,766 participants (case = 896, control = 870), a significant reduction in TC concentration (WMD: −0.10 mmol/L, 95% CI: −0.17 to −0.02, *p* <0.05) (Figure 9A) was observed, with slight effect heterogeneity (*I*^2^ =14.6%, *p* = 0.233). Regarding funnel plot symmetry and Egger's test, *p* = 0.907 (Figure 10A).

A pooled meta-analysis including 34 arms with 1,750 participants (case = 887, control = 863) evaluated the effect of IF on TG level (Figure 9B). The WMD using a fixed-effects model was −0.09 mmol/L (95% CI: −0.13 to −0.04, *p* <0.05), which indicates significant TG reduction. There was no evidence of effect heterogeneity among the studies (*I*^2^ =5.5%, *p* = 0.377). Regarding funnel plot symmetry and Egger's test, *p* = 0.868 (Figure 10B).

A pooled meta-analysis including 36 arms with 1,850 participants (case = 943, control = 907) found no tangible effect of IF on LDL-C concentration (WMD: −0.056 mmol/L, 95% CI: −0.114 to 0.003, *p* > 0.05) (Figure 9C). There was also no evidence of effect heterogeneity among the studies (*I*^2^ =0.0%, *p* = 0.967). Regarding funnel plot symmetry and Egger's test, *p* = 0.214 (Figure 10C).

There were 36 RCTs involving 1,852 participants (case =943, control = 909) that evaluated the effect of IF on changes in HDL-C. Data pooling showed no significant changes in HDL-C level (WMD: −0.01 mmol/L, 95% CI: −0.04 to 0.01, *p* > 0.05) (Figure 9D), with no significant heterogeneity (*I*^2^ = 0.0%, *p* = 0.858). Regarding funnel plot symmetry and Egger's test, *p* = 0.711 (Figure 10D).

### Sensitivity Analysis

In order to determine the impact of each individual study on the effect index, we used a sensitivity analysis in our meta-analysis. Finally, we did not observe the significant effects of any individual study (Supplementary Figures 1–4).

## Discussion

In this study, 46 articles with 55 arms were systematically reviewed to evaluate the effects of IF on CMRFs. The pooled analysis showed that IF had significantly reduced body composition (weight, WC, FM, and BMI), BP (SBP, DBP), lipid panel (TC, TG), and improved glycemic control by reducing FBG, Fins, and HOMA-IR; however, it did not affect the HbA1c level and lipid profile (LDL-C and HDL-C).

### Comparison With Other Systematic Reviews

Compared with the previous six systematic reviews of IF on cardiac metabolic risk-related indicators, the meta-analysis has obvious distinction and added novel value: First, there were three RCT (76–78) and three non-RCT articles (79–81) in the previous meta-analysis. We included only RCTs that represented the highest level of scientific evidence. Second, there were 30 (77), 19 (79), 28 (80), 10 (76), 15 (81), and 15 articles (78) included in the previous six meta-analyses on IF. We pooled the results of 46 articles to estimate the impact of IF on CMRFs. Third, some studies have focused on metabolic drivers, such as abnormal adipocytokine (81), dysglycemia (76, 77), blood pressure (79), blood lipids (80), and inflammatory factors (78). However, we provided the most comprehensive CMRF indicators, including body weight, blood sugar, blood lipids, and blood pressure. Finally, we also included studies with participants who had a range of subject types, including obese, sedentary, overweight, and healthy-weight adults, as well as those with type 2 diabetes and non-alcoholic liver disease. In addition, we included baseline levels of changes, rather than absolute levels of metabolic indicators, so as to assess the true impact of IF on these parameters, regardless of the health status of participants.

### Effects of Body Composition

Overall, in terms of body composition, there was a significant positive correlation between BMI and weight loss during IF (i.e., the higher the starting BMI, the greater the weight loss during the fasting period). This suggests that IF may be more effective for people with a higher BMI. The results for the effect of IF on body composition were similar to those obtained in a previous meta-analysis, by (82), which involved 11 trials that found that TRF was effective in promoting weight loss and reducing FBG compared to not limiting meal times approaches. In addition, IER was more effective in reducing weight than a regular control diet. Moreover, it was also more effective in reducing FM level than CER (17). In a meta-analysis on religious fasting (83), it was found that overweight participants had a greater reduction in weight and percentage of fat than normal people. A recent meta-analysis of RCTs showed that ADF effectively lowered body composition (BMI, weight, and FM) in overweight adults within 6 months compared to the control group (84). However, in another meta-analysis of 12 RCTs, researchers confirmed that lean mass was relatively conserved in the IF group, and no significant weight reduction was identified (59). In addition, a recent study by Lowe et al. (22) on 16:8 time-restricted eating (TRE), an IF plan encouraging the consumption of all dietary intake within an 8-h eating window, demonstrated that IF does not play a significant role in weight loss in the absence of controlled food intake. In turn, it may lead to a reduction in muscle mass. There were no significant differences in FM, Fins, glucose level, HbA1C, or blood lipids between the TRE and control groups. The results of Lowe's study are contrary to the results of most studies related to fasting. This may have been brought about by the time window of fasting. Meantime, our meta-analysis is also included in this study, but a comprehensive assessment of IF in body composition is beneficial.

### Effects of Glycemic Control

O'Keefe et al. (85) found that IF habits can improve glucose metabolism as well as reduce abdominal fat accumulation, free radical production, inflammation, and the risks of diabetes, CVD, cancer, and neurodegenerative diseases. After 12 h of fasting, insulin levels drop, liver glycogen reserves are depleted, and the body begins to absorb fatty acids from fat cells to replace glucose for combustion, which improves insulin sensitivity (12, 86). In reality, the effectiveness of IF and energy restriction on the levels of FBG, insulin level, and HOMA-IR in human participants remains inconsistent.

In a recent meta-analysis of RCTs, IF-induced FBG, Fins, and HOMA-IR were more effectively controlled than with a normal diet or CER, which was in keeping with the findings of our study. Moreover, there was also a decrease in HbA1c, but not statistically significant. Compared with our findings, this analysis has a significant effect on reducing FBG and fibrin levels. However, these analyses were based on only 35 arms, whereas we included 55 arms (77). In contrast, in some studies, no significant association was found between IF and FBG (8).

Furthermore, the impaired activity of the IGF-1 axis could be the basis of numerous metabolic, biochemical, and functional alterations that characterize aging and disease (87). In a recent meta-analysis of IF and energy-restricted diets on IGF-1 levels, Rahmani et al. revealed that only fasting regimens and energy restriction to ≤ 50% normal required daily energy intake resulted in significantly reduced levels of plasma IGF-1 in human participants (76).

### Effects of Glycemic Control

In the lipid module analysis, the findings of a recent meta-analysis of the effects of IF and energy-limited diet on blood lipids were similar to those in our systematic review. The results showed that, compared with the control group, IF significantly reduced the concentration of TC and TG, but had no meaningful effects on HDL-C concentration (80). However, the two meta-analyses are contradictory in terms of LDL-C levels. The lack of standardization of blood lipid measurement time in individual studies may lead to changes in LDL-C concentrations in some studies. In healthy adults, the longer the week of fasting, the higher the TC, LDL-C, and other indicators (88). This meta-analysis contains the energy limitation of uninterrupted time, which may be the main reason for the difference. Although we did not observe a significant effect of IF on HDL-C concentration, some previous studies have demonstrated the effect of dietary intervention on this lipid marker. Moro et al. demonstrated a small increase in HDL-C concentrations in people who exercised for 8 weeks as part of a normal-calorie diet (43).

Previous studies have shown that IF is not only beneficial in reducing the production of free radicals or weight loss; it also has several health benefits (7, 89–91). IF can cause an evolutionarily conservative adaptive cellular response, improve blood glucose regulation, enhance anti-stress ability, and inhibit inflammation between and within organs. During fasting, cells activate pathways that enhance the body's defense against oxidation and metabolic stress, as well as remove or repair damaged molecules. IF improves the healthy physiological indexes related to metabolism, which lays the foundation for improving the metabolic characteristics of the animal (91, 92). Fat is the main energy source for cells and is stored in the adipose tissue in the form of TGs after meals. During fasting, TGs are broken down into fatty acids and glycerol, which are used to provide energy consumption by the organism (93).

Although not discussed in this study, we found further interesting indicators (leptin and adiponectin secreted by adipocytes) that were used in weight control studies. A meta-analysis by Varkaneh Kord et al. (81) revealed that there was a significant reduction in serum leptin and a non-significant reduction in serum adiponectin following IF. This provides more reference indicators for the effect of IF on metabolic risk.

### Effects of Blood Pressure

Blood pressure is an important metabolic index of CVD; thus, any viable mechanism to reduce or manage vascular indices, such as blood pressure, warrants detailed investigation. The reduction of SBP (2.14 mmHg) was observed in our meta-analysis compared with the control group. In the current meta-analysis, IF was slightly more effective in reducing SBP (3.26 mmHg) than energy-restricting diets (1.09 mmHg). The reason for this difference was that, in our study, IF was mainly used in the intervention group, while CER and daily diet were used in the control group.

The available data show that the increase in vascular oxidative stress and vascular inflammation is not only the mechanism of endothelial dysfunction but also the key to the pathogenesis of hypertension (94, 95); and an empirical study showed that fasting reduces hypertension (12, 89, 96). The mechanism of the decreased blood pressure may be the increase in parasympathetic activity due to the brain-derived neurotrophic factor, increased norepinephrine excretion by the kidneys, and increased sensitivity of natriuretic peptides and insulin (97). Cardiovascular health benefits do not persist after termination of the IF diet. After cessation of the diet, the blood pressure values return to their initial values (98).

Although many studies have shown that IF is beneficial to human health and suitable for a wide range of metabolic diseases, this dietary model has rarely been promoted in practice. The main reason for this lack of implementation is that people are accustomed to eating three meals a day. When changing to an IF program, some people feel hungry, irritable, and lose concentration. In addition, IF has side effects in humans. Previous studies have shown that the exercise level of athletes during fasting is extremely decreased due to hypoglycemia (99); there are also disturbances in sleep architecture. A recent study by Lowe et al. (22) demonstrated that in the absence of controlled food intake, IF will lead to a reduction in muscle mass. Incorrect fasting time windows can lead to gastrointestinal diseases. In unhealthy people, using fasting therapy can lead to a decline in physical fitness. Finally, IF includes different fasting methods, time windows, and energy limitations, which require consultation with a dietitian or doctor to adopt an appropriate fasting schedule to ensure the patient's nutritional needs, while gradually reducing the time window for daily eating to prevent side effects, and simultaneously providing continuous counseling and education.

There are some limitations to this meta-analysis. As this is the case with some studies, some of the included ones have a small sample size, and there are several studies with a high risk of bias. Second, the number of long-term studies conducted is very limited, and larger long-term trials with a longer duration are needed to understand the effects of IF on weight loss and long-term weight management. Moreover, different types of IF have different characteristics in various metabolic diseases, and we did not analyze each of them individually. Finally, although IF has a variety of components, a comparison with other types of IF could not be conducted due to the lack of RCT research on religious fasting and lack of data on other kinds of fasting.

## Conclusion

This systematic review has demonstrated that IF may improve body composition (weight, WC, FM, and BMI) and moderate BP, TC, TG, and blood glucose, but there may be no difference regarding the LDL-C, HDL-C, and HbA1c levels; components of CMRFs are also risk factors for the development of diabetes and CVDs. Therefore, high-quality and long-term RCTs are needed to provide data on the persistence of the effect and to strengthen the certainty of the evidence.

## Data Availability Statement

The original contributions presented in the study are included in the article/Supplementary Material, further inquiries can be directed to the corresponding authors.

## Author Contributions

YX and YG designed the manuscript. FY wrote the manuscript. FY, XLiu, and CL searched databases performed the selection of studies. YX and XP revised the manuscript. XLi, LT, and JS critically evaluated the review and commented on it. SY, RZ, NA, and XY contributed in revised version. All authors approved the manuscript for publication.

## Funding

This work was supported by the National Key R&D Program of China (grants 2018YFC1704900 and 2018YFC1704901) and the CACMS Innovation Fund (grant number CI2021A00919).

## Conflict of Interest

The authors declare that the research was conducted in the absence of any commercial or financial relationships that could be construed as a potential conflict of interest.

## Publisher's Note

All claims expressed in this article are solely those of the authors and do not necessarily represent those of their affiliated organizations, or those of the publisher, the editors and the reviewers. Any product that may be evaluated in this article, or claim that may be made by its manufacturer, is not guaranteed or endorsed by the publisher.

## Abbreviations

IF: Intermittent fasting
CMRFs: cardiometabolic risk factors
RCTs: randomized controlled trials
CVDs: cardiovascular diseases
PRISMA: Preferred Reporting Items for Systematic Reviews and Meta-Analyses
CI: confidence interval
WMD: weighted mean difference
ADF: Alternate-day fasting
AMDF: alternate-modified-day fasting
CR: Caloric restriction
TRF: time-restricted feeding
TRE: time-restricted eating
IER: Intermittent energy restriction
CER: Continuous energy restriction
WC: waist circumference
FM: fat mass
BMI: body mass index
SBP: systolic blood pressure
DBP: diastolic blood pressure
TC: total cholesterol
TG: triglyceride
HDL: high-density lipoprotein
LDL: low-density lipoprotein
FBG: fasting blood glucose
Fins: fasting insulin
HbA1c: glycosylated hemoglobin
HOMA-IR: insulin resistance
MetS: metabolic syndrome
VLCD: very low-calorie diet
IECR: Intermittent energy and carbohydrate restriction.
