# Supplementary material for: Effect of Epidemic Intermittent Fasting on Cardiometabolic Risk Factors: A Systematic Review and Meta-Analysis of Randomized Controlled Trials
Source: Front Nutr. 2021 Oct 18;8:669325. doi: 10.3389/fnut.2021.669325 (PMC8558421; doi:10.3389/fnut.2021.669325)
Supplement: Supplementary file 1 [file Data_Sheet_1.docx]

# Supplementary Material

## 1 Supplementary Tables

**Table S1. Search strategy.**

| PubMed | Cochrane Library | Embase |
| --- | --- | --- |
| ((("Fasting"[Mesh]) OR (((((((((((intermittent fasting[Title/Abstract]) OR (time-restricted fasting[Title/Abstract])) OR (time-restricted feeding[Title/Abstract])) OR (time-restricted diet[Title/Abstract])) OR (alternate fasting[Title/Abstract])) OR (alternate-day modified fasting[Title/Abstract])) OR (alternate-day fasting[Title/Abstract])) OR (reduced meal frequency[Title/Abstract])) OR (periodic diet[Title/Abstract])) OR (periodic fasting[Title/Abstract])) OR (Intermittent energy restriction))) AND (((((((((((((((((((((metabolic syndrome[Title/Abstract]) OR (cardiometabolic risk factors[Title/Abstract])) OR (body composition[Title/Abstract])) OR (weight[Title/Abstract])) OR (waist circumference[Title/Abstract])) OR (fasting glucose[Title/Abstract])) OR (diabetes mellitus[Title/Abstract])) OR (obesity[Title/Abstract])) OR (insulin[Title/Abstract])) OR (hypertension[Title/Abstract])) OR (dyslipidemia[Title/Abstract])) OR (low-density lipoprotein[Title/Abstract])) OR (blood pressure[Title/Abstract])) OR (high-density lipoprotein[Title/Abstract])) OR (triglycerides[Title/Abstract])) OR (blood lipids[Title/Abstract])) OR (lipid profile[Title/Abstract])) OR (Metabolic Cardiovascular[Title/Abstract])) OR (Cardiovascular Syndrome[Title/Abstract])) OR (Insulin Resistance[Title/Abstract])) OR (Dysmetabolic Syndrome[Title/Abstract]))) AND (randomized controlled trial[Publication Type] OR randomized[Title/Abstract] OR placebo[Title/Abstract]) | (MeSH descriptor: [Fasting] explode all trees OR (intermittent fasting):ti,ab,kw OR (time-restricted fasting):ti,ab,kw OR (time-restricted feeding):ti,ab,kw OR (time-restricted diet):ti,ab,kw OR (alternate fasting):ti,ab,kw OR (alternate-day modified fasting):ti,ab,kw OR (alternate-day fasting):ti,ab,kw OR (reduced meal frequency):ti,ab,kw OR (periodic diet):ti,ab,kw OR (periodic fasting):ti,ab,kw OR (intermittent energy restriction):ti,ab,kw) AND (MeSH descriptor: [Metabolic Syndrome] explode all trees OR (metabolic syndrome x):ti,ab,kw OR (cardio metabolic risk factors):ti,ab,kw OR (body composition):ti,ab,kw OR (weight):ti,ab,kw OR (waist circumference):ti,ab,kw OR (glucose):ti,ab,kw OR (diabetes mellitus):ti,ab,kw OR (obesity):ti,ab,kw OR (insulin):ti,ab,kw OR (hypertension):ti,ab,kw OR (dyslipidemia):ti,ab,kw OR (low density lipoprotein):ti,ab,kw OR (high-density lipoprotein):ti,ab,kw OR (blood pressure):ti,ab,kw OR (blood pressure):ti,ab,kw OR (blood lipids):ti,ab,kw OR (lipid profile):ti,ab,kw OR (metabolic cardiovascular):ti,ab,kw OR (cardiovascular syndrome):ti,ab,kw OR (insulin resistance):ti,ab,kw OR (dysmetabolic syndrome):ti,ab,kw) | ('intermittent fasting'/exp OR 'intermittent fasting':ti,ab OR 'time-restricted fasting':ti,ab OR 'time-restricted feeding':ti,ab OR 'time-restricted diet':ti,ab OR 'alternate fasting':ti,ab OR 'alternate-day modified fasting':ti,ab OR 'alternate-day fasting':ti,ab OR 'reduced meal frequency':ti,ab OR 'periodic diet':ti,ab OR periodic fasting':ti,ab OR 'intermittent energy restriction':ti,ab) AND (metabolic syndrome x'/exp OR 'cardio metabolic risk factors':ti,ab OR 'body composition':ti,ab OR 'weight':ti,ab OR 'waist circumference':ti,ab OR 'glucose':ti,ab OR 'diabetes mellitus':ti,ab OR 'obesity':ti,ab OR 'insulin':ti,ab OR 'hypertension':ti,ab OR 'dyslipidemia':ti,ab OR 'low density lipoprotein':ti,ab OR 'high-density lipoprotein':ti,ab OR 'blood pressure':ti,ab OR 'blood pressure':ti,ab OR 'blood lipids':ti,ab OR 'lipid profile':ti,ab OR 'metabolic cardiovascular':ti,ab OR 'cardiovascular syndrome':ti,ab OR 'insulin resistance':ti,ab OR 'dysmetabolic syndrome':ti,ab) AND ('random':ti,ab OR 'placebo':ti,ab OR 'double-blind':ti,ab OR 'randomized controlled trial':ti,ab) |

## 2 Supplementary Figures


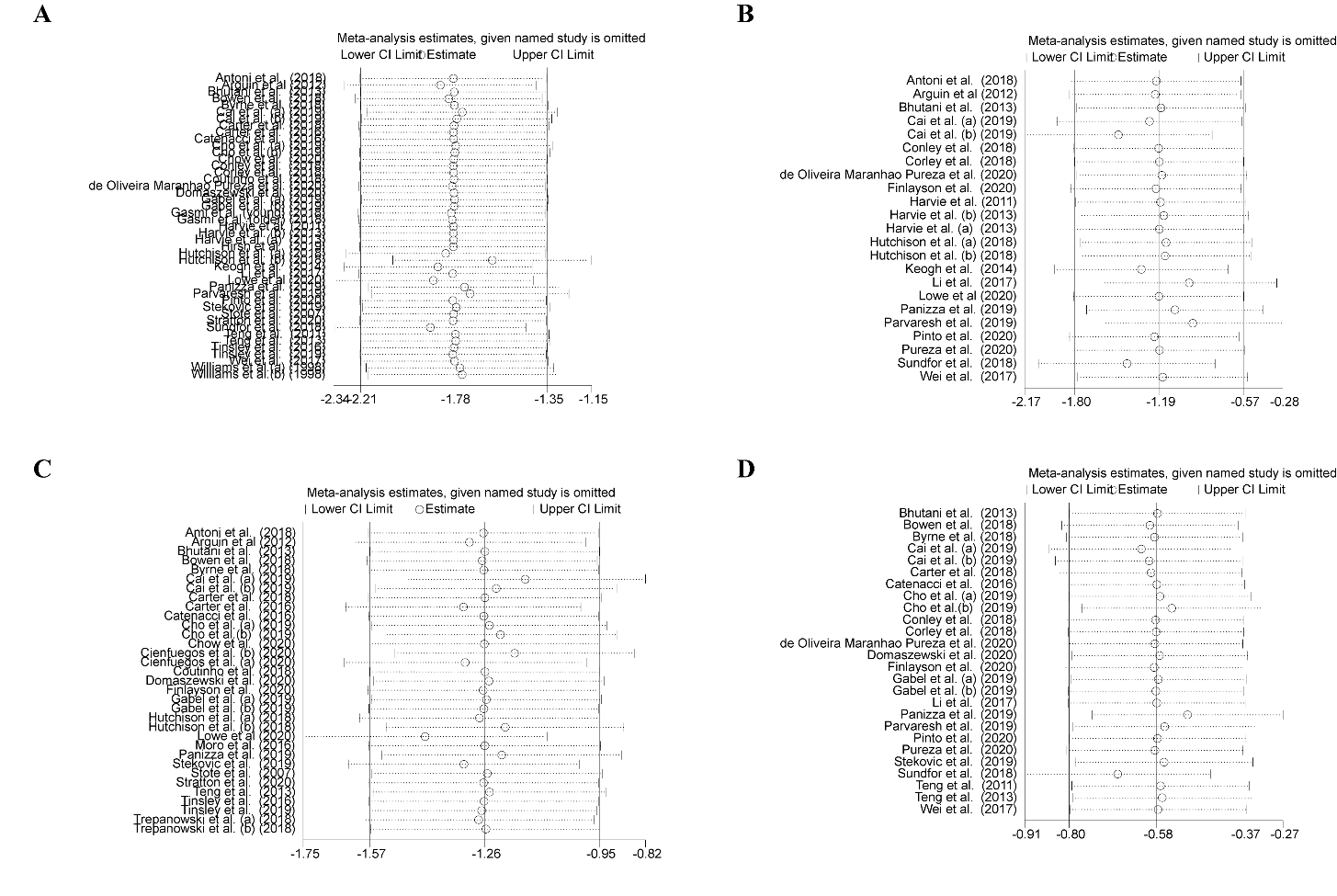


**Figure S1.** Sensitivity analysis observed no significant effect of intermittent fasting on body composition (A) Weight, (B) WC, (C) FM, (D) BMI.


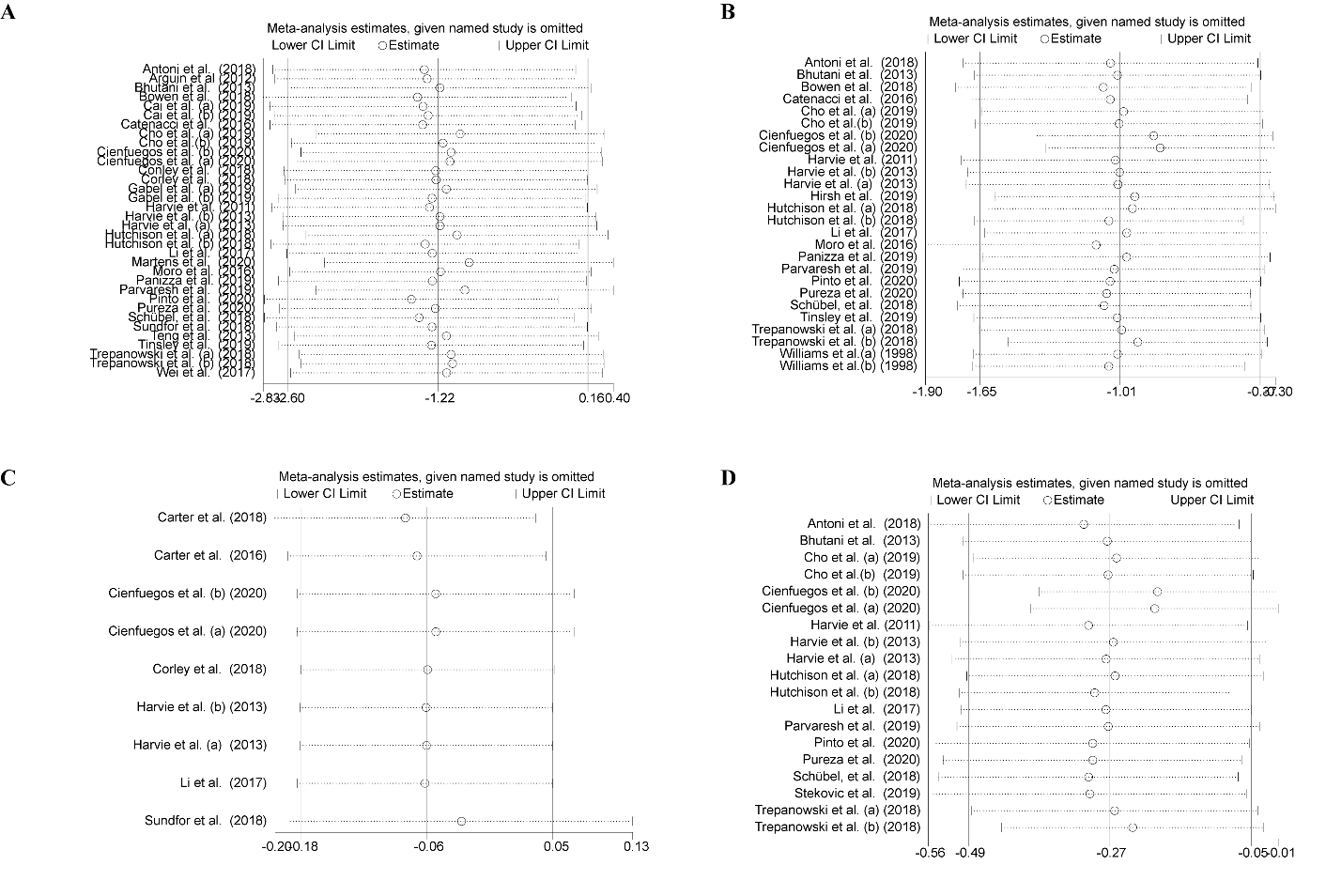


**Figure S2.** Sensitivity analysis observed no significant effect of intermittent fasting on glycemic control (A) FBG, (B) Fins, (C) HbA1c, (D) HOMA-IR.


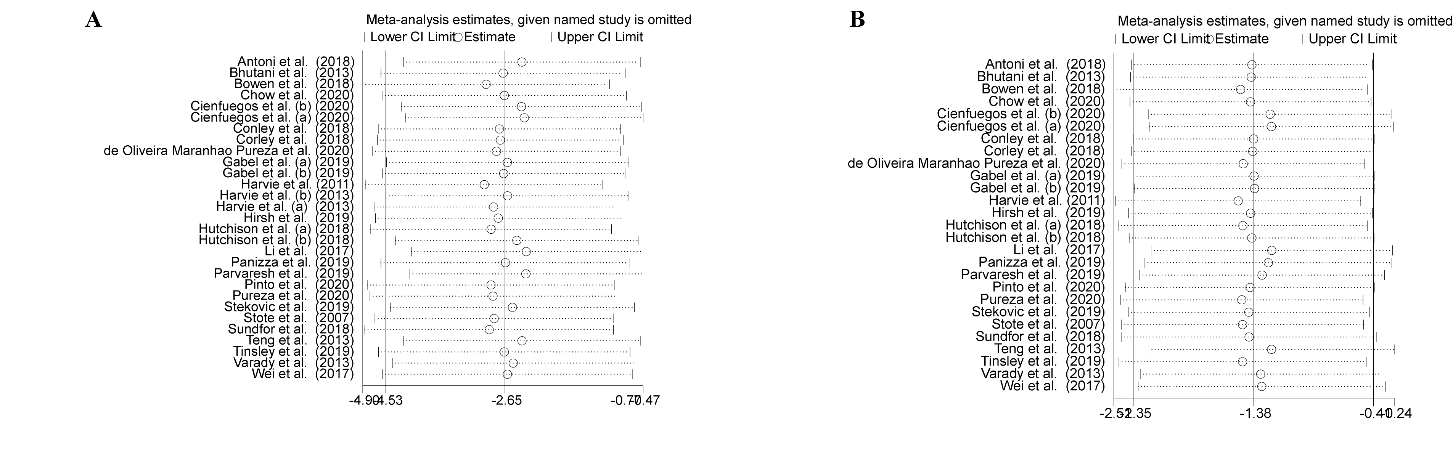


**Figure S3.** Sensitivity analysis observed no significant effect of intermittent fasting on blood pressure (A) SBP and (B) DBP.


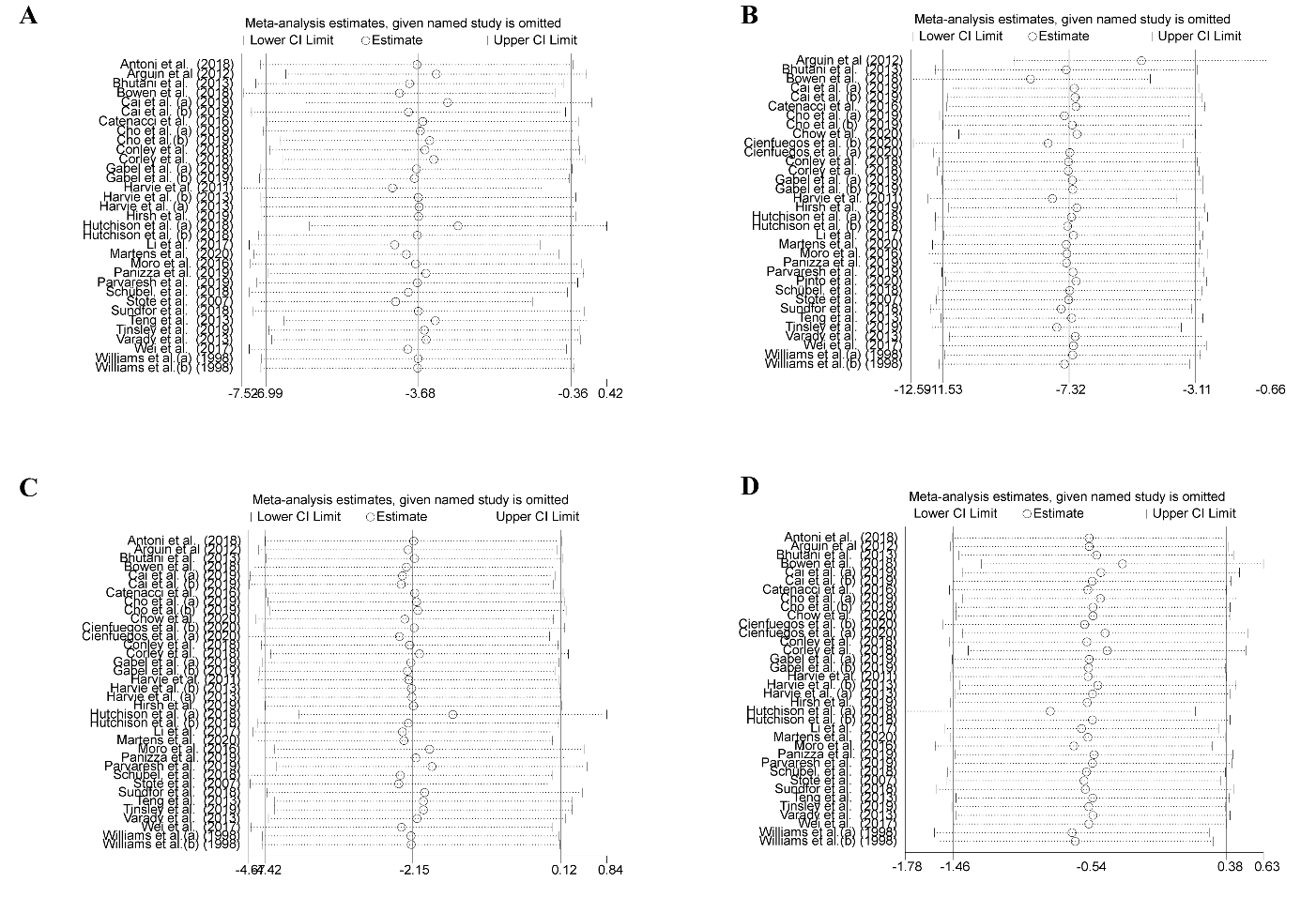


**Figure S4.** Sensitivity analysis observed no significant effect of intermittent fasting on lipid panel (A) TC, (B) TG, (C) LDL-C, (D) HDL-C.
