# Supplementary material for: Effect of Epidemic Intermittent Fasting on Cardiometabolic Risk Factors: A Systematic Review and Meta-Analysis of Randomized Controlled Trials
Source: Front Nutr. 2021 Oct 18;8:669325. doi: 10.3389/fnut.2021.669325 (PMC8558421; doi:10.3389/fnut.2021.669325)
Supplement: Supplementary file 2 [file Table_2.docx]

**Supplementary Table 2. Characteristics of studies included in the meta-analysis**

| Study and  publication year | Country | RCT design (blinding) | Total  sample  (case:  control) | Participants | Intervention and intervention details | Control | Duration | Mean age | Sex (F: M) | Outcomes |
| --- | --- | --- | --- | --- | --- | --- | --- | --- | --- | --- |
| Chow et al. (2020) [1] | USA | A randomized clinical trial | 11:9 | People with overweight or obesity | TRF: 11:8 hour window, with unrestricted eating within the window | Unrestricted (non-TRE) control | 12w | 46.5 ±12.4 | 9:2 | weight, FM, SBP, DBP, TG, LDL-C, HDL-C |
| Cienfuegos et al. (2020) (a) [2] | USA | A randomized controlled trial | 16:14 | Adults with obesity | 4-hour TRF: eating only between 3 and 7 pm (without having to count calories) | The control group continued their usual diet pattern with no meal timing restrictions | 8w | 47 ± 8 | 14:2 | FM, SBP, DBP, TG, LDL-C, HDL-C, FBG, Fins, HbA1c, HOMA-IR |
| Cienfuegos et al. (2020) (b) [2] | USA | A randomized controlled trial | 19:14 | Adults with obesity | 6-hour TRF: eating only between 1 and 7 pm (without having to count calories) | The control group continued their usual diet pattern with no meal timing restrictions | 8w | 47 ± 13.8 | 18:1 | FM, SBP, DBP, TG, LDL-C, HDL-C, FBG, Fins, HbA1c, HOMA-IR |
| de Oliveira Maranhao Pureza et al. (2020) [3] | Brasil | A randomized, parallel, controlled clinical trial | 31:27 | Obesity people | TRF and a hypoenergetic diet: meals only in a 12-hour feeding window and fasting for the other 12 hours. | Hypo energetic diet | 12w | 31.03 ±7.16 | NA | weight, WC, BMI, SBP, DBP |
| Domaszewski et al. (2020) [4] | Poland | A randomized clinical trial | 25:20 | Overweight women over 60 years of age | Experimental group involved  completely abstaining from food for 16 hours a day, from 8 pm to 12 am (the next day) | Followed their previous eating plan | 6w | 65 ± 4 | 25f | weight, FM, BMI |
| Finlayson et al. (2020) [5] | USA | A parallel-group controlled-feeding randomized controlled trial | 24:22 | Overweight and obesity Women | IER: alternating ad libitum and 75% energy restriction days | CER | 12w | 34 ± 10 | 24f | WC, FM, BMI, |
| Lowe et al. (2020) [6] | USA | A randomized clinical trial | 59:57 | Adults with obesity | TRF: Eat 8 hours a day and fast the rest of the day | Consistent meal timing | 12w | 46.8 ±10.8 | 24:35 | weight, WC, FM |
| Martens et al. (2020) [7] | USA | A randomized controlled crossover trial | 14:10 | Healthy middle-aged and older men and postmenopausal women | TRF: Eat 8 hours a day and fast the rest of the day | Chronic calorie restriction | 6w | 66 ± 6.92 | 7:5 | TC, TG, LDL-C, HDL-C, FBG |
| Pinto et al. (2020) [8] | UK | A parallel-arm randomized controlled trial | 21:22 | Non-smoking men and　women | Short-term effects of IER: 48 hours, 600 kcal/day, followed by 5-day  healthy eating advice | CER: 500 kcal/day, healthy eating advice | 4w | 50 ± 12 | 15:6 | weight, WC, BMI, SBP, DBP, TG, FBG, Fins, HOMA-IR |
| Pureza et al. (2020) [9] | Brazil | A randomized, parallel, controlled trial | 31:27 | Women with obesity | Hypoenergetic diet with TRF. women were instructed to eat only  during a 12 hour and fasted during the other 12 hours | A diet with the same energy  restriction but without TRF | 21d | 31.8 ± 7.25 | 31f | Weight, WC, BMI, SBP, DBP, FBG, Fins, HOMA-IR |
| Stratton et al. (2020) [10] | USA | A randomized controlled trial | 13:13 | Active males | TRF: 8 hours eating window, 25% caloric deficit, 1.8 g/kg/day protein, and body resistance training | Normal diet and body resistance training | 4w | 22.9 ± 3.6 | 13m | weight, FM |
| Cai et al. (a) (2019) [11] | China | A randomized clinical trial | 90:79 | Adults with nonalcoholic fatty liver disease (NAFLD) | ADF: 25% baseline energy needs, mealtime between 12.00 p.m. and 2.00 p.m | Control group | 12w | 35.50±4.417 | 60:35 | weight, WC, FM, BMI, TC, TG, LDL-C, HDL-C, FBG, Fins |
| Cai et al. (b) (2019) [11] | China | A randomized clinical trial | 95:79 | Adults with nonalcoholic fatty liver disease (NAFLD) | TRF: 16:8 fasting window | Control group | 12w | 33.56 ± 6.23 | 66:29 | weight, WC, FM, BMI, TC, TG, LDL-C, HDL-C, FBG, Fins |
| Cho et al. (a) (2019) [12] | Korea | A randomized, controlled, parallel-arm diet trial | 9:9 | Adults with with overweight or obesity | ADF and exercise | Continued their regular  eating and exercise habits | 8w | 34.5 ± 5.7 | 4:5 | weight, FM, BMI, TC, TG, LDL-C, HDL-C, FBG, Fins, HOMA-IR |
| Cho et al. (b) (2019) [12] | Korea | A randomized, controlled, parallel-arm diet trial | 8:9 | Adults with with overweight or obesity | ADF | CER | 8w | 33.5 ± 5 | 6:2 | weight, FM, BMI, TC, TG, LDL-C, HDL-C, FBG, Fins, HOMA-IR |
| Gabel et al. (a) (2019) [13] | USA | Secondary analysis of a study | 11:17 | Individuals with overweight and obesity | ADF: participants consumed 25% of their baseline energy needs at lunch  (between 12 and 2 pm) | Control  group | 12m | 43±9.95 | 9:2 | weight, FM, BMI, SBP, DBP, TC, TG, LDL-C, HDL-C, FBG |
| Gabel et al. (b) (2019) [13] | USA | Secondary analysis of a study | 11:15 | Individuals with overweight and obesity | ADF: participants consumed 25% of their baseline energy needs at lunch  (between 12 and 2 pm) | CR: consumed 75% baseline energy | 12m | 43±9.95 | 9:2 | weight, FM, BMI, SBP, DBP, TC, TG, LDL-C, HDL-C, FBG |
| Hirsh et al. (2019) [14] | USA | A randomized clinical trial | 10:12 | Overweight individuals | Nutrition  program  group: two fasting days of balanced shake and dietary supplements, 5 days of habitual diet | Habitual diet | 52d | 43.4±13 | 8:2 | weight, SBP, DBP, TC, TG, LDL-C, HDL-C, Fins |
| Panizza et al. (2019) [15] | USA | A randomized active comparator pilot study | 30:30 | BMI 25–40 kg/m^2^, VAT ≥ 90 cm for men and women | IER and a Mediterranean diet: 2 consecutive days with 70% energy restriction and 5 days of a Mediterranean diet | Dietary Approaches to Stop Hypertension diet | 12w | 48.4±4.7 | 21:9 | weight, WC, FM, BMI, SBP, DBP, TC, TG, LDL-C, HDL-C, FBG, Fins |
| Parvaresh et al. (2019) [16] | Iran | A single-center, randomized clinical trial | 35:34 | Patients with MetS and overweight | ADF: 25% of the individual's energy needs | CR | 8w | 44.6±9.08 | 14:20 | weight, WC, BMI, SBP, DBP, TC, TG, LDL-C, HDL-C, FBG, Fins, HOMA-IR |
| Stekovic et al. (2019) [17] | Austria | An embedded  randomized controlled trial | 30:30 | Healthy study participants | ADF: eat every secondday ad libitum, refrain from calorie intake on the fast days | Ad libitum number of meals | 4w | 48 | 17:12 | weight, FM, BMI, SBP, DBP, HOMA-IR |
| Tinsley et al. (2019) [18] | USA | A randomized controlled trial | 13:14 | Healthy females | TRF: consume all calories between 12 and 8 pm each day | Control diet | 8w | 22.1 ± 7.27 | 13f | weight, FM, SBP, DBP, TC, TG, LDL-C, HDL-C, FBG, Fins |
| Hutchison et al. (2019) (a) [19] | Australia | A randomized controlled trial | 25:26 | Overweight women | IF70: an IF diet at 70% of calculated baseline energy requirements per week | Dietary restriction (DF70) | 8w | 49 ± 10 | 25f | weight, WC, SBP, DBP, TC, TG, LDL-C, HDL-C, FBG, Fins, HOMA-IR |
| Hutchison et al. (2019) (b) [19] | Australia | A randomized controlled trial | 25:12 | Overweight women | IF100: an IF diet at 100% of calculated baseline energy requirements per week | Continuous energy intake at 100 % of baseline energy | 8w | 51 ± 10 | 25f | weight, WC, SBP, DBP, TC, TG, LDL-C, HDL-C, FBG, Fins, HOMA-IR |
| Antoni et al. (2018) [20] | UK | A randomized, parallel-arm trial | 15:12 | Individuals with overweight and obesity | IER:  25% of the energy requirements for two consecutive days. On the remaining 5 normal days | CER | 7d | 45±15.49 | 7:8 | weight, WC, FM, SBP, DBP, TC, LDL-C, HDL-C, FBG, Fins, HOMA-IR |
| Bowen et al. (2018) [21] | Australia | A randomized parallel study | 82:81 | Adults with overweight and obesity | ADF + Daily energy restriction (DER); 3 days of ADF, 3 days of alternate DER, and one ad libitum day | Daily energy restriction | 16w | 40.0 ± 8.3 | 67:15 | weight, FM, BMI, SBP, DBP, TC, TG, LDL-C, HDL-C, FBG, Fins |
| Byrne et al. (2018) [22] | Australia | A single-center, parallel-group, randomized controlled trial | 26:25 | Males with obesity | IER: alternating ad libitum and 75% energy restriction days | CER | 16w | 39.9± 9.2 | 26m | weight, FM, BMI |
| Carter et al. (2018) [23] | Australia | A randomized noninferiority trial | 70:67 | Adults with type 2 diabetes who were  overweight or obese | IER: 500-600 kcal/day, followed for 2 nonconsecutive days per week (their usual diet for the other 5 days) | CER | 12m | 61±9 | 39:31 | weight, FM, BMI, HbA1c |
| Conley et al. (2018) [24] | Australia | A single-center, parallel-group randomized controlled trial | 11:12 | Veterans: males with a BMI greater than or equal to 30 kg/m^2^ and stable weight | IER: 2 non-consecutive days per week (restrict calorie intake to 600 calories) and eat ad libitum on the remaining 5 days | Standard energy-restricted diet | 3m | 68 ± 2.7 | 11m | weight, WC, BMI, SBP, DBP, TC, TG, LDL-C, HDL-C, FBG |
| Corley et al. (2018) [25] | Australia | A randomized controlled trial | 19:18 | Participants with type 2 diabetes who were taking medication for diabetes | Non-consecutive days caloric restriction: 5:2 schedule a VLCD for 2 days per week | CR | 12w | 58 (42 to 74) | 8:11 | weight, WC, FM, BMI, SBP, DBP, TC, TG, LDL-C, HDL-C, FBG, HbA1c |
| Coutinho et al. (2018) [26] | Norway | A randomized controlled trial | 14:14 | Adults with obesity | IER: 3 non-consecutive days (followed a commercial very low-calorie diet (550 and 660 kcal/day for women and men, respectively) | CER: followed a low-calorie diet | 12w | 39.4±11.0 | 10:4 | weight, FM |
| Gasmi et al. (2018) (young) [27] | Italy | A randomized controlled trial | 10:10 | Young men | TRF: young and older were asked to fast for 2 days separated by 48 hours (Monday and Thursday) for 3 months (February, March, April) | Normal meals | 12w | 26.90±1.97 | 10m | weight |
| Gasmi et al. (2018) (old) [27] | Italy | A randomized controlled trial | 10:10 | Aged men | TRF: young and older were asked to fast for 2 days separated by 48 hours (Monday and Thursday) for 3 months (February, March, April) | Normal meals | 12w | 51.60±5.87 | 10m | weight |
| Schübel, et al. (2018) [28] | Germany | A randomized controlled trial | 49:49 | Men and women with overweight and obesity | IER: 5:2 diet (2 days with 75% energy deficit and 5 days without energy restriction) | No advice to restrict energy | 12w | 49.4 ± 9.0 | 24:25 | TC, TG, LDL-C, HDL-C, FBG, Fins, HOMA-IR |
| Sundfor et al. (2018) [29] | Norway | A randomized controlled clinical trial | 54:58 | Men and women with overweight and obesity | IER: 5:2 diet | CER | 6m | 49.9±10.1 | 26:28 | weight, WC, BMI, SBP, DBP, TC, TG, LDL-C, HDL-C, FBG, HbA1c |
| Trepanowski et al. (a) (2018) [30] | USA | A randomized controlled trial | 25:29 | Men and women with overweight and obesity | ADF: repeatedly alternate between consuming 25% of energy needs over 24-hour | CR | 24w | 46 ± 10 | 22:3 | FM, FBG, Fins, HOMA-IR |
| Trepanowski et al. (b) (2018) [30] | USA | A randomized controlled trial | 25:25 | Men and women with overweight and obesity | ADF: repeatedly alternate between consuming 25% of energy needs over 24-hour | Consumed 100% of energy needs every day | 24w | 46 ± 10 | 22:3 | FM, FBG, Fins, HOMA-IR |
| Li et al. (2017) [31] | Germany | A randomized controlled clinical pilot study | 23:23 | Persons with a manifest and treated type 2 diabetes | A 7-day fasting program (an initial fasting program followed a Mediterranean diet) | A Mediterranean diet | 4m | 64.7 ± 7.0 | NA | weight, WC, BMI, SBP, DBP, TC, TG, LDL-C, HDL-C, FBG, Fins, HbA1c, HOMA-IR |
| Wei et al. (2017) [32] | USA | A randomized crossover  design | 52:48 | Healthy participants | Fasting-mimicking diet: a plant-based diet designed to attain fasting-like effects | Unrestricted diet | 3m | 43.3 ± 11.7 | 33:19 | weight, WC, BMI, SBP, DBP, TC, TG, LDL-C, HDL-C, FBG |
| Carter et al. (2016) [33] | Australia | A parallel randomized controlled trial | 31:32 | Obesity adults with type 2 diabetes mellitus; BP of <160/100 mmHg | IER: an ER of 1670-2500kJ/day for 2 days each week, and the remaining 5 days included habitual eating | CER | 12w | 61 ± 7.5 | 17:14 | weight, FM, HbA1c |
| Catenacci et al. (2016) [34] | USA | A randomized pilot study | 13:12 | Individuals with obesity | ADF: zero-calories | Caloric restriction (2400 kcal/day) | 8w | 39.6±9.5 | 9:3 | weight FM, BMI, TC, TG, LDL-C, HDL-C, FBG, Fins, |
| Moro et al. (2016) [35] | Italy | A randomized controlled trial | 17:17 | Resistance-trained males | TRF: participants consumed 100% of energy needs in an 8-hour | Normal  diet group | 8w | 29.94 ± 4.07 | 17m | FM, TC, TG, LDL-C, HDL-C, FBG, Fins |
| Tinsley et al. (2016) [36] | USA | A randomized controlled trial | 10:8 | Generally healthy, recreationally active men | Resistance training and TRF: consuming all calories within a four-hour period,  4 days per week. Resistance training program  was performed 3 days per week | Resistance training and normal diet | 8w | 22.9 ± 4.1 | 10m | weight, FM |
| Keogh et al. (2014) [37] | Australia | A parallel, randomized controlled trial | 19:17 | Women with overweight or obesity | IER: 1-week normal diet followed by 1 week of energy restriction | CER | 52w | 59.5 ± 8.7 | 19f | weight, WC |
| Harvie et al. (2014) (b) [38] | USA | A single-center, randomized  study | 37:40 | Women with overweight or obesity | IECR: restrict energy and carbohydrates on 2 consecutive days each week and Mediterranean-type diet for the remaining 5 days of the week | Daily energy restriction | 3m | 45.6±8.3 | 37f | weight, WC, SBP, TC, LDL-C, HDL-C, FBG, Fins, HbA1c, HOMA-IR |
| Harvie et al. (a) (2014) [38] | USA | A single-center, randomized  study | 38:40 | Women with overweight or obesity | IECR and ad libitum protein and fat | Daily energy restriction | 3m | 48.6 ±7.3 | 38f | weight, WC, SBP, TC, LDL-C, HDL-C, FBG, Fins, HbA1c, HOMA-IR |
| Teng et al. (2013) [39] | Malaysia | A randomized controlled trial | 28:28 | Healthy (non-diabetic and no history of　cardiovascular diseases) Malay men | Fasting calorie restriction | Maintain their present lifestyle | 6w | 59.6±5.4 | 28m | weight, FM, BMI, SBP, DBP, TC, TG, LDL-C, HDL-C, FBG |
| Varady et al. (2013) [40] | USA | A randomized, controlled, parallel-arm feeding  trial | 15:15 | Healthy people | ADF: 25% of their baseline energy needs on the fast day and then ate ad libitum on each alternating feed day | Ad libitum | 12w | 47±7.74 | 10:5 | SBP, DBP, TC, TG, LDL-C, HDL-C |
| Bhutani et al. (2013) [41] | USA | A randomized, controlled, parallel-arm feeding trial | 25:16 | Adults with obesity | A 4-week controlled  feeding period: 25% of their baseline energy needs on the ‘‘fast day’’ and an 8-week self-selected “feeding period” | Ad libitum number of meals | 12w | 42 ± 10 | 24:1 | weight, WC, FM, BMI, SBP, DBP, TC, TG, LDL-C, HDL-C, FBG, Fins, HOMA-IR |
| Arguin et al. (2012) [42] | USA | A randomized pilot study | 12:10 | Postmenopausal women with sedentary obesity | IF: food was self-selected with dietitian supervision on macronutrient composition (55%, 30%, and 15% of energy intake from carbohydrates, fats, and proteins, respectively | Continuous diet | 30w | 60.5 ±6.0 | 12f | weight, WC, FM, TC, TG, LDL-C, HDL-C, FBG |
| Harvie et al. (2011) [43] | USA | A randomized trial | 53:54 | Premenopausal women with overweight | IER: 25% restriction delivered as a VLCD for 2 days per week, with no restrictions on the other 5 days of the week. | CER | 6m | 40 ±14.1 | 53f | weight, WC, SBP, DBP, TC, TG, LDL-C, HDL-C, FBG, Fins, HOMA-IR |
| Teng et al. (2011) [44] | USA | A randomized controlled trial | 13:12 | Healthy Malay men | Fasting calorie restriction: reduce daily energy intake by 300-500 kcal/day and fast two days a week for three months | Maintenance of present lifestyle | 12w | 59.3 ± 3.4 | 13m | weight, BMI |
| Stote et al. (2007) [45] | USA | A randomized crossover design | Total (15) | Healthy, normal-weight, middle-aged adults | 1 meal/d | 3  meals/d | 8w | 45 ±2.71 | 10:5 | weight, FM, SBP, DBP, TC, TG, LDL-C, HDL-C, FBG |
| Williams et al. (1998) (a) [46] | USA | A parallel  Arms | 18:18 | T2DM patients | IER (1 day/  week): 400–  600 kcal/day  on fast day and  1500–1800 kcal/  day on feed day | CER:1500–  1800 kcal/day  every day | 20w | 51 ± 8 | 9:9 | weight, TC, TG, LDL-C, HDL-C, Fins |
| Williams et al. (1998) (b) [46] | USA | A parallel  Arms | 18:18 | T2DM patients | IER (5 days/  week): 400–  600 kcal/day on  fast day every  5 weeks and  1500–1800 kcal/  day on feed days | CER:1500–  1800 kcal/day  every day | 20w | 50 ± 9 | 11:7 | weight, TC, TG, LDL-C, HDL-C, Fins |
|  |  |  |  |  |  |  |  |  |  |  |
|  |  |  |  |  |  |  |  |  |  |  |

Reference

[1] L.S. Chow, E.N.C. Manoogian, A. Alvear, J.G. Fleischer, H. Thor, K. Dietsche, Q. Wang, J.S. Hodges, N. Esch, S. Malaeb, T. Harindhanavudhi, K.S. Nair, S. Panda, and D.G. Mashek, Time‐Restricted Eating Effects on Body Composition and Metabolic Measures in Humans who are Overweight: A Feasibility Study. Obesity 28 (2020) 860-869. doi:10.1002/oby.22756

[2] S. Cienfuegos, K. Gabel, F. Kalam, M. Ezpeleta, E. Wiseman, V. Pavlou, S. Lin, M.L. Oliveira, and K.A. Varady, Effects of 4- and 6-h Time-Restricted Feeding on Weight and Cardiometabolic Health: A Randomized Controlled Trial in Adults with Obesity. Cell Metab 32 (2020) 366-378 e3. doi:10.1016/j.cmet.2020.06.018

[3] I.R. de Oliveira Maranhão Pureza, A.E. da Silva Junior, D.R. Silva Praxedes, L.G. Lessa Vasconcelos, M. de Lima Macena, I.S. Vieira de Melo, T.M. de Menezes Toledo Florêncio, and N.B. Bueno, Effects of time-restricted feeding on body weight, body composition and vital signs in low-income women with obesity: A 12-month randomized clinical trial. Clin Nutr (2020). doi:10.1016/j.clnu.2020.06.036

[4] P. Domaszewski, M. Konieczny, P. Pakosz, D. Bączkowicz, and E. Sadowska-Krępa, Effect of a Six-Week Intermittent Fasting Intervention Program on the Composition of the Human Body in Women over 60 Years of Age. International Journal of Environmental Research and Public Health 17 (2020). doi:10.3390/ijerph17114138

[5] G. Finlayson, J. Blundell, K. Varady, M. Hopkins, C. Gibbons, J. Turicchi, P. Oustric, N. Casanova, and K. Beaulieu, Matched Weight Loss Through Intermittent or Continuous Energy Restriction Does Not Lead To Compensatory Increases in Appetite and Eating Behavior in a Randomized Controlled Trial in Women with Overweight and Obesity. The Journal of Nutrition 150 (2020) 623-633. doi:10.1093/jn/nxz296

[6] D.A. Lowe, N. Wu, L. Rohdin-Bibby, A.H. Moore, N. Kelly, Y.E. Liu, E. Philip, E. Vittinghoff, S.B. Heymsfield, J.E. Olgin, J.A. Shepherd, and E.J. Weiss, Effects of Time-Restricted Eating on Weight Loss and Other Metabolic Parameters in Women and Men With Overweight and Obesity: The TREAT Randomized Clinical Trial. JAMA Intern Med (2020). doi:10.1001/jamainternmed.2020.4153

[7] C.R. Martens, M.J. Rossman, M.R. Mazzo, L.R. Jankowski, E.E. Nagy, B.A. Denman, J.J. Richey, S.A. Johnson, B.P. Ziemba, Y. Wang, C.M. Peterson, M. Chonchol, and D.R. Seals, Short-term time-restricted feeding is safe and feasible in non-obese healthy midlife and older adults. Geroscience 42 (2020) 667-686. doi:10.1007/s11357-020-00156-6

[8] A.M. Pinto, C. Bordoli, L.P. Buckner, C. Kim, P.C. Kaplan, I.M. Del Arenal, E.J. Jeffcock, and W.L. Hall, Intermittent energy restriction is comparable to continuous energy restriction for cardiometabolic health in adults with central obesity: A randomized controlled trial; the Met-IER study. Clin Nutr 39 (2020) 1753-1763. doi:10.1016/j.clnu.2019.07.014

[9] I. Pureza, I.S.V. Melo, M.L. Macena, D.R.S. Praxedes, L.G.L. Vasconcelos, A.E. Silva-Júnior, T. Florêncio, and N.B. Bueno, Acute effects of time-restricted feeding in low-income women with obesity placed on hypoenergetic diets: Randomized trial. Nutrition 77 (2020) 110796. doi:10.1016/j.nut.2020.110796

[10] M.T. Stratton, G.M. Tinsley, M.G. Alesi, G.M. Hester, A.A. Olmos, P.R. Serafini, A.S. Modjeski, G.T. Mangine, K. King, S.N. Savage, A.T. Webb, and T.A. VanDusseldorp, Four Weeks of Time-Restricted Feeding Combined with Resistance Training Does Not Differentially Influence Measures of Body Composition, Muscle Performance, Resting Energy Expenditure, and Blood Biomarkers. Nutrients 12 (2020). doi:10.3390/nu12041126

[11] H. Cai, Y.L. Qin, Z.Y. Shi, J.H. Chen, M.J. Zeng, W. Zhou, R.Q. Chen, and Z.Y. Chen, Effects of alternate-day fasting on body weight and dyslipidaemia in patients with non-alcoholic fatty liver disease: a randomised controlled trial. BMC Gastroenterol 19 (2019) 219. doi:10.1186/s12876-019-1132-8

[12] A.R. Cho, J.Y. Moon, S. Kim, K.Y. An, M. Oh, J.Y. Jeon, D.H. Jung, M.H. Choi, and J.W. Lee, Effects of alternate day fasting and exercise on cholesterol metabolism in overweight or obese adults: A pilot randomized controlled trial. Metabolism 93 (2019) 52-60. doi:10.1016/j.metabol.2019.01.002

[13] K. Gabel, C.M. Kroeger, J.F. Trepanowski, K.K. Hoddy, S. Cienfuegos, F. Kalam, and K.A. Varady, Differential Effects of Alternate-Day Fasting Versus Daily Calorie Restriction on Insulin Resistance. Obesity (Silver Spring) 27 (2019) 1443-1450. doi:10.1002/oby.22564

[14] S.P. Hirsh, M. Pons, S.V. Joyal, and A.G. Swick, Avoiding holiday seasonal weight gain with nutrient-supported intermittent energy restriction: a pilot study. J Nutr Sci 8 (2019) e11. doi:10.1017/jns.2019.8

[15] C.E. Panizza, U. Lim, K.M. Yonemori, K.D. Cassel, L.R. Wilkens, M.N. Harvie, G. Maskarinec, E.J. Delp, J.W. Lampe, J.A. Shepherd, L. Le Marchand, and C.J. Boushey, Effects of Intermittent Energy Restriction Combined with a Mediterranean Diet on Reducing Visceral Adiposity: A Randomized Active Comparator Pilot Study. Nutrients 11 (2019). doi:10.3390/nu11061386

[16] A. Parvaresh, R. Razavi, B. Abbasi, K. Yaghoobloo, A. Hassanzadeh, N. Mohammadifard, S.M. Safavi, A. Hadi, and C.C.T. Clark, Modified alternate-day fasting vs. calorie restriction in the treatment of patients with metabolic syndrome: A randomized clinical trial. Complementary Therapies in Medicine 47 (2019). doi:10.1016/j.ctim.2019.08.021

[17] S. Stekovic, S.J. Hofer, N. Tripolt, M.A. Aon, P. Royer, L. Pein, J.T. Stadler, T. Pendl, B. Prietl, J. Url, S. Schroeder, J. Tadic, T. Eisenberg, C. Magnes, M. Stumpe, E. Zuegner, N. Bordag, R. Riedl, A. Schmidt, E. Kolesnik, N. Verheyen, A. Springer, T. Madl, F. Sinner, R. de Cabo, G. Kroemer, B. Obermayer-Pietsch, J. Dengjel, H. Sourij, T.R. Pieber, and F. Madeo, Alternate Day Fasting Improves Physiological and Molecular Markers of Aging in Healthy, Non-obese Humans. Cell Metabolism 30 (2019) 462-476.e6. doi:10.1016/j.cmet.2019.07.016

[18] G.M. Tinsley, M.L. Moore, A.J. Graybeal, A. Paoli, Y. Kim, J.U. Gonzales, J.R. Harry, T.A. VanDusseldorp, D.N. Kennedy, and M.R. Cruz, Time-restricted feeding plus resistance training in active females: a randomized trial. Am J Clin Nutr 110 (2019) 628-640. doi:10.1093/ajcn/nqz126

[19] A.T. Hutchison, B. Liu, R.E. Wood, A.D. Vincent, C.H. Thompson, N.J. O'Callaghan, G.A. Wittert, and L.K. Heilbronn, Effects of Intermittent Versus Continuous Energy Intakes on Insulin Sensitivity and Metabolic Risk in Women with Overweight. Obesity (Silver Spring) 27 (2019) 50-58. doi:10.1002/oby.22345

[20] R. Antoni, K.L. Johnston, A.L. Collins, and M.D. Robertson, Intermittent v. continuous energy restriction: differential effects on postprandial glucose and lipid metabolism following matched weight loss in overweight/obese participants. British Journal of Nutrition 119 (2018) 507-516. doi:10.1017/s0007114517003890

[21] J. Bowen, E. Brindal, G. James-Martin, and M. Noakes, Randomized Trial of a High Protein, Partial Meal Replacement Program with or without Alternate Day Fasting: Similar Effects on Weight Loss, Retention Status, Nutritional, Metabolic, and Behavioral Outcomes. Nutrients 10 (2018). doi:10.3390/nu10091145

[22] N.M. Byrne, A. Sainsbury, N.A. King, A.P. Hills, and R.E. Wood, Intermittent energy restriction improves weight loss efficiency in obese men: the MATADOR study. Int J Obes (Lond) 42 (2018) 129-138. doi:10.1038/ijo.2017.206

[23] S. Carter, P.M. Clifton, and J.B. Keogh, Effect of Intermittent Compared With Continuous Energy Restricted Diet on Glycemic Control in Patients With Type 2 Diabetes: A Randomized Noninferiority Trial. JAMA Netw Open 1 (2018) e180756. doi:10.1001/jamanetworkopen.2018.0756

[24] M. Conley, L. Le Fevre, C. Haywood, and J. Proietto, Is two days of intermittent energy restriction per week a feasible weight loss approach in obese males? A randomised pilot study. Nutr Diet 75 (2018) 65-72. doi:10.1111/1747-0080.12372

[25] B.T. Corley, R.W. Carroll, R.M. Hall, M. Weatherall, A. Parry-Strong, and J.D. Krebs, Intermittent fasting in Type 2 diabetes mellitus and the risk of hypoglycaemia: a randomized controlled trial. Diabet Med 35 (2018) 588-594. doi:10.1111/dme.13595

[26] S.R. Coutinho, E.H. Halset, S. Gåsbakk, J.F. Rehfeld, B. Kulseng, H. Truby, and C. Martins, Compensatory mechanisms activated with intermittent energy restriction: A randomized control trial. Clin Nutr 37 (2018) 815-823. doi:10.1016/j.clnu.2017.04.002

[27] M. Gasmi, M. Sellami, J. Denham, J. Padulo, G. Kuvacic, W. Selmi, and R. Khalifa, Time-restricted feeding influences immune responses without compromising muscle performance in older men. Nutrition 51-52 (2018) 29-37. doi:10.1016/j.nut.2017.12.014

[28] R. Schübel, J. Nattenmüller, D. Sookthai, T. Nonnenmacher, M.E. Graf, L. Riedl, C.L. Schlett, O. von Stackelberg, T. Johnson, D. Nabers, R. Kirsten, M. Kratz, H.-U. Kauczor, C.M. Ulrich, R. Kaaks, and T. Kühn, Effects of intermittent and continuous calorie restriction on body weight and metabolism over 50 wk: a randomized controlled trial. The American Journal of Clinical Nutrition 108 (2018) 933-945. doi:10.1093/ajcn/nqy196

[29] T.M. Sundfør, M. Svendsen, and S. Tonstad, Effect of intermittent versus continuous energy restriction on weight loss, maintenance and cardiometabolic risk: A randomized 1-year trial. Nutr Metab Cardiovasc Dis 28 (2018) 698-706. doi:10.1016/j.numecd.2018.03.009

[30] J.F. Trepanowski, C.M. Kroeger, A. Barnosky, M. Klempel, S. Bhutani, K.K. Hoddy, J. Rood, E. Ravussin, and K.A. Varady, Effects of alternate-day fasting or daily calorie restriction on body composition, fat distribution, and circulating adipokines: Secondary analysis of a randomized controlled trial. Clinical Nutrition 37 (2018) 1871-1878. doi:10.1016/j.clnu.2017.11.018

[31] C. Li, B. Sadraie, N. Steckhan, C. Kessler, R. Stange, M. Jeitler, and A. Michalsen, Effects of A One-week Fasting Therapy in Patients with Type-2 Diabetes Mellitus and Metabolic Syndrome – A Randomized Controlled Explorative Study. Experimental and Clinical Endocrinology & Diabetes 125 (2017) 618-624. doi:10.1055/s-0043-101700

[32] M. Wei, S. Brandhorst, M. Shelehchi, H. Mirzaei, C. Cheng, J. Budniak, S. Groshen, W. Mack, E. Guen, S. Di Biase, P. Cohen, T. Morgan, T. Dorff, K. Hong, A. Michalsen, A. Laviano, and V. Longo, Fasting-mimicking diet and markers/risk factors for aging, diabetes, cancer, and cardiovascular disease. Science translational medicine 9 (2017). doi:10.1126/scitranslmed.aai8700

[33] S. Carter, P.M. Clifton, and J.B. Keogh, The effects of intermittent compared to continuous energy restriction on glycaemic control in type 2 diabetes; a pragmatic pilot trial. Diabetes Research and Clinical Practice 122 (2016) 106-112. doi:10.1016/j.diabres.2016.10.010

[34] V.A. Catenacci, Z. Pan, D. Ostendorf, S. Brannon, W.S. Gozansky, M.P. Mattson, B. Martin, P.S. MacLean, E.L. Melanson, and W. Troy Donahoo, A randomized pilot study comparing zero-calorie alternate-day fasting to daily caloric restriction in adults with obesity. Obesity (Silver Spring) 24 (2016) 1874-83. doi:10.1002/oby.21581

[35] T. Moro, G. Tinsley, A. Bianco, G. Marcolin, Q. Pacelli, G. Battaglia, A. Palma, P. Gentil, M. Neri, and A. Paoli, Effects of eight weeks of time-restricted feeding (16/8) on basal metabolism, maximal strength, body composition, inflammation, and cardiovascular risk factors in resistance-trained males. Journal of translational medicine 14 (2016) 290. doi:10.1186/s12967-016-1044-0

[36] G.M. Tinsley, J.S. Forsse, N.K. Butler, A. Paoli, A.A. Bane, P.M. La Bounty, G.B. Morgan, and P.W. Grandjean, Time-restricted feeding in young men performing resistance training: A randomized controlled trial. European Journal of Sport Science 17 (2016) 200-207. doi:10.1080/17461391.2016.1223173

[37] J.B. Keogh, E. Pedersen, K.S. Petersen, and P.M. Clifton, Effects of intermittent compared to continuous energy restriction on short-term weight loss and long-term weight loss maintenance. Clinical Obesity 4 (2014) 150-156. doi:10.1111/cob.12052

[38] M. Harvie, C. Wright, M. Pegington, D. McMullan, E. Mitchell, B. Martin, R.G. Cutler, G. Evans, S. Whiteside, S. Maudsley, S. Camandola, R. Wang, O.D. Carlson, J.M. Egan, M.P. Mattson, and A. Howell, The effect of intermittent energy and carbohydrate restrictionv. daily energy restriction on weight loss and metabolic disease risk markers in overweight women. British Journal of Nutrition 110 (2013) 1534-1547. doi:10.1017/s0007114513000792

[39] N.I. Teng, S. Shahar, N.F. Rajab, Z.A. Manaf, M.H. Johari, and W.Z. Ngah, Improvement of metabolic parameters in healthy older adult men following a fasting calorie restriction intervention. Aging Male 16 (2013) 177-83. doi:10.3109/13685538.2013.832191

[40] K. Varady, S. Bhutani, M. Klempel, C. Kroeger, J. Trepanowski, J. Haus, K. Hoddy, and Y. Calvo, Alternate day fasting for weight loss in normal weight and overweight subjects: a randomized controlled trial. Nutr J 12 (2013) 146. doi:10.1186/1475-2891-12-146

[41] S. Bhutani, M.C. Klempel, C.M. Kroeger, J.F. Trepanowski, and K.A. Varady, Alternate day fasting and endurance exercise combine to reduce body weight and favorably alter plasma lipids in obese humans. Obesity (Silver Spring) 21 (2013) 1370-9. doi:10.1002/oby.20353

[42] H. Arguin, I.J. Dionne, M. Sénéchal, D.R. Bouchard, A.C. Carpentier, J.L. Ardilouze, A. Tremblay, C. Leblanc, and M. Brochu, Short- and long-term effects of continuous versus intermittent restrictive diet approaches on body composition and the metabolic profile in overweight and obese postmenopausal women: a pilot study. Menopause 19 (2012) 870-6. doi:10.1097/gme.0b013e318250a287

[43] M.N. Harvie, M. Pegington, M.P. Mattson, J. Frystyk, B. Dillon, G. Evans, J. Cuzick, S.A. Jebb, B. Martin, R.G. Cutler, T.G. Son, S. Maudsley, O.D. Carlson, J.M. Egan, A. Flyvbjerg, and A. Howell, The effects of intermittent or continuous energy restriction on weight loss and metabolic disease risk markers: a randomized trial in young overweight women. Int J Obes (Lond) 35 (2011) 714-27. doi:10.1038/ijo.2010.171

[44] N.I. Teng, S. Shahar, Z.A. Manaf, S.K. Das, C.S. Taha, and W.Z. Ngah, Efficacy of fasting calorie restriction on quality of life among aging men. Physiol Behav 104 (2011) 1059-64. doi:10.1016/j.physbeh.2011.07.007

[45] K.S. Stote, D.J. Baer, K. Spears, D.R. Paul, G.K. Harris, W.V. Rumpler, P. Strycula, S.S. Najjar, L. Ferrucci, D.K. Ingram, D.L. Longo, and M.P. Mattson, A controlled trial of reduced meal frequency without caloric restriction in healthy, normal-weight, middle-aged adults. The American journal of clinical nutrition 85 (2007) 981-988.

[46] K.V. Williams, M.L. Mullen, D.E. Kelley, and R.R. Wing, The effect of short periods of caloric restriction on weight loss and glycemic control in type 2 diabetes. Diabetes Care 21 (1998) 2-8. doi:10.2337/diacare.21.1.2
